# Taking Advantage of the Morpheein Behavior of Peroxiredoxin in Bionanotechnology

**DOI:** 10.1021/acs.bioconjchem.0c00621

**Published:** 2021-01-07

**Authors:** Matteo Ardini, Andrea Bellelli, David L. Williams, Luana Di Leandro, Francesco Giansanti, Annamaria Cimini, Rodolfo Ippoliti, Francesco Angelucci

**Affiliations:** § Department of Life, Health, and Environmental Sciences, University of L’Aquila, Piazzale Salvatore Tommasi 1, 67100 L’Aquila, Italy; # Department of Biochemical Sciences “A. Rossi Fanelli”, University of Roma “Sapienza”, Piazzale Aldo Moro 5, 00185 Roma, Italy; ⊥ Department of Microbial Pathogens and Immunity, Rush University Medical Center, Chicago, Illinois 60612, United States

## Abstract

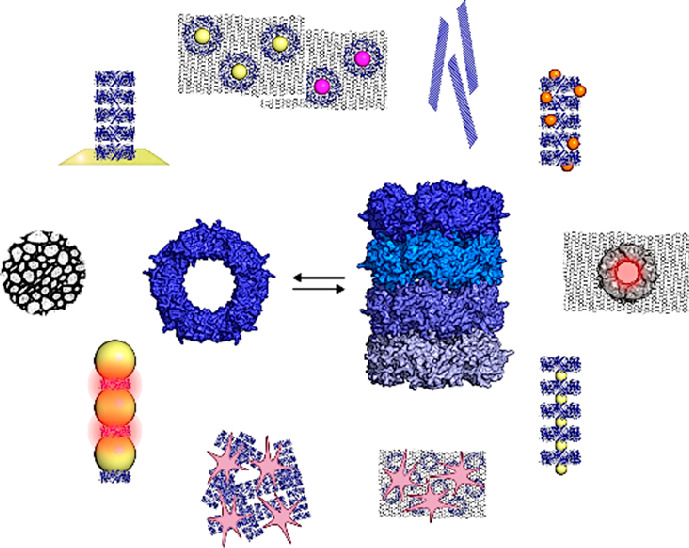

Morpheeins are proteins that reversibly assemble into different oligomers, whose architectures are governed by conformational changes of the subunits. This property could be utilized in bionanotechnology where the building of nanometric and new high-ordered structures is required. By capitalizing on the adaptability of morpheeins to create patterned structures and exploiting their inborn affinity toward inorganic and living matter, “bottom-up” creation of nanostructures could be achieved using a single protein building block, which may be useful as such or as scaffolds for more complex materials. Peroxiredoxins represent the paradigm of a morpheein that can be applied to bionanotechnology. This review describes the structural and functional transitions that peroxiredoxins undergo to form high-order oligomers, e.g., rings, tubes, particles, and catenanes, and reports on the chemical and genetic engineering approaches to employ them in the generation of responsive nanostructures and nanodevices. The usefulness of the morpheeins’ behavior is emphasized, supporting their use in future applications.

## 1 Introduction

Advances in biochemistry have shed light on unusual proteins that defy the dogma “one sequence, one structure, one function”, i.e., chameleonic proteins, intrinsically disordered proteins, metamorphic proteins, and morpheeins.^1^ These biological polymers can fold into multiple secondary and tertiary structures and as morpheeins self-assemble into several quaternary complexes starting from a single protomer.^2^ Due to different conformational states of the protomer, often related to the motions associated with the protein primary functions, morpheeins naturally undergo self-assembly into different homo-oligomers, which may have distinct activities directly correlated to the exposure of hidden surfaces and associated with recognition of new ligands. Thus, morpheein behavior can be seen a stratagem adopted by nature to generate new protein functions or tune pre-existing ones by changing the oligomeric state.^3^ Furthermore, the equilibrium between morpheein oligomers can be shifted by changes in temperature, pH, ionic strength, and redox potential as well as single point mutations,^2^ encouraging the use of the resulting supramolecular architectures as templates and scaffolds in bionanotechnology.

Bionanotechnology relies on the application of knowledge of the structural and functional properties of biomolecules obtained by *in vivo* and/or *in vitro* studies to create new architectures at the nanoscale showing chemical and/or physical properties useful for practical purposes: the possibility of building different supramolecular structures relies on the natural occurrence of biological building blocks characterized by different shapes. Among these, proteins are the most adaptable due to their unique collection of chemical functionalities and their variability in shape with respect to other, simpler biomolecules such as RNA, DNA, and peptides. Proteins can establish a plethora of specific covalent and noncovalent interactions exploiting their intrinsic and unique chemical versatility with affinity toward nanomaterials including zero-dimensional (0D) inorganic^4^ and organic nanoparticles (NPs),^5^ two-dimensional (2D) lattices such as graphene, graphene oxide (GO) and reduced GO (rGO),^6^ as well as one-dimensional (1D) carbon nanotubes.^7^ Moreover, gold (Au) and silver (Ag) nanomaterials are very suitable for conjugation with proteins due to their affinity for binding to the −SH and −S–CH_3_ groups of cysteine and methionine.^8^ In addition, such chemical versatility can be improved by genetic engineering or chemical cross-linking, where *ad hoc* insertion of functionalities offers endless possibilities of protein engineering.^9−11^ This inherent capability is further refined upon folding of the protein polymer into the tertiary and quaternary structure: during folding some amino acidic functional groups are exposed at the protein surface, creating distinct patches of chemical functionalities, and the subsequent oligomerization process patterns them in a regular manner onto the resulting 3D tertiary and quaternary assembly. The oligomers usually possess higher structural stability and enhanced biological functions with respect to single subunits as well as increased binding affinity for enzyme substrates and receptor ligands.^12^ Further, proteins are prone to anisotropic hierarchical self-assembly under mild conditions, forming regular 1–100 nm large supramolecular homo- and hetero-oligomers,^13^ including filaments,^14^ rings,^15^ tubes,^16^ cages,^17−20^ catenanes,^21^ and knots.^22^ In some cases, protein superstructures have been used as scaffolds to direct the polymerization of more durable materials, or to synthesize mixed polymers endowed with specific properties, e.g., electrical conductivity.^23^ The success of such protein assemblies starts to impact even at commercial level, for instance, next-generation sequencing of nucleic acids using protein nanopore-based devices by the Oxford Nanopore Technologies.^24^ However, even though many natural proteins have evolved on their own to self-associate, in general the lack of control over the assembly and the need of structural stability *in vitro* limits their applications. To overcome this, different approaches, including the matching rotational symmetry method, interface design, and directed evolution, have been explored to create several oligomeric assemblies; these approaches are engineering-intensive for protein surface and dependent on the precision of the design.^25^ However, using a single building block to create different high-order oligomeric protein assemblies remains a challenge.^26^

**Figure 1:**
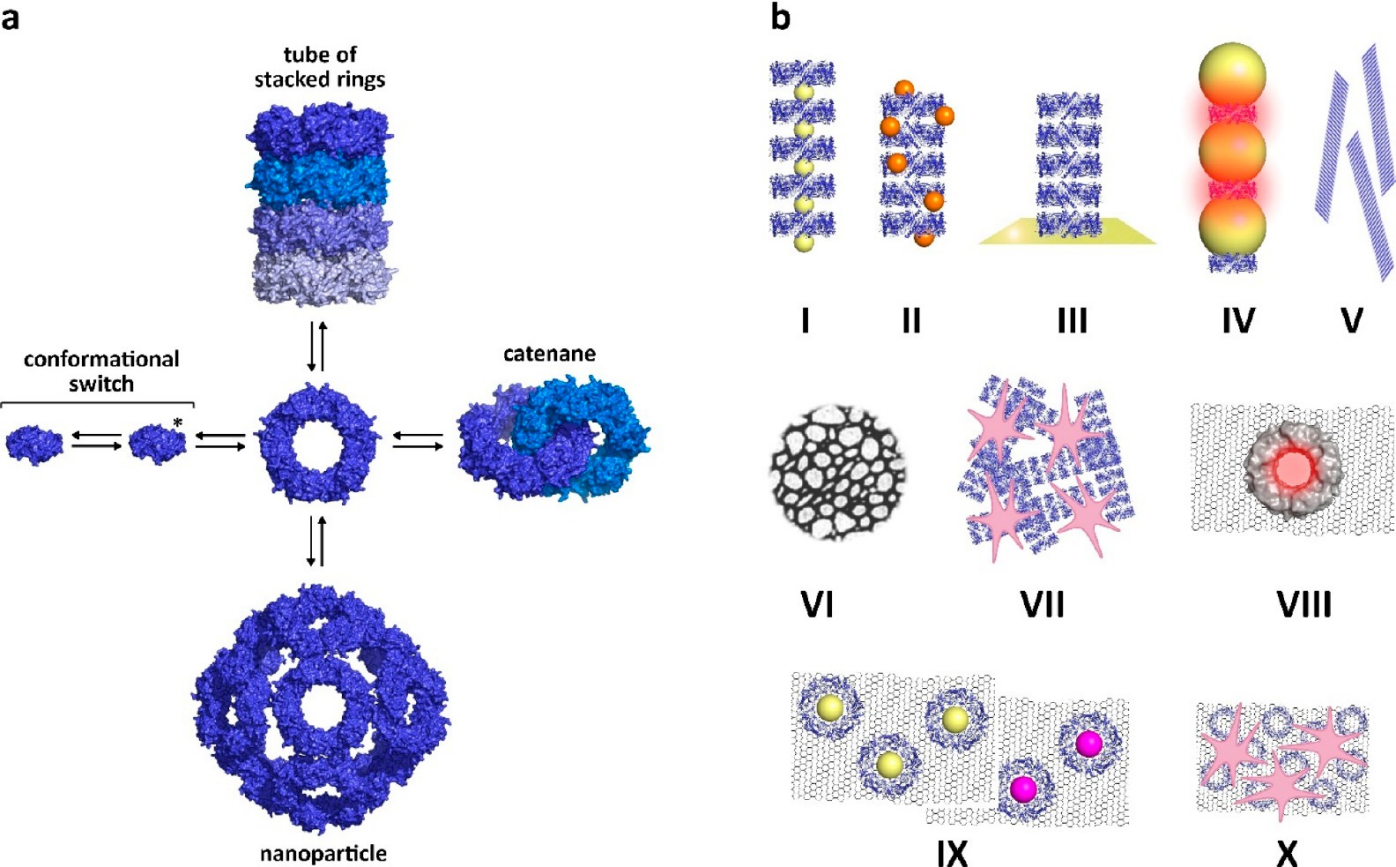
Prx morpheeins in bionanotechnology. (a) Typical 2-Cys Prx morpheeins conformational switch upon chemical and physical stimuli or genetic engineering to form patterned homo-oligomers. (b) Prx morpheeins can be exploited to obtain (I) polarizable gold NPs arrays, (II) polarizable iron NPs arrays, (III) gold-tethered tubes and rings, (IV) plasmonic gold NPs arrays, (V) semiconductive nanoribbons for transistors, (VI) rGO composites, (VII) biocompatible scaffolds, (VIII) plasmonic nanopores arrays, (IX) gold and palladium NPs-doped rGO composites, and (X) biocompatible rGO scaffolds.

Looking for such “ready-to-use” biological “tools” for bionanotechnology, morpheeins are worth considering, as they can avoid the afore-mentioned problems if the structural shift of the building block that gives rise to different nanometric architectures can be controlled. Members of the typical 2-Cys peroxiredoxin subfamily (Prx) are naturally able to assemble in various high-order homo-oligomers such as rings, tubes of stacked rings, cage-like particles, and even catenanes^27−30^ (Figure 1a). Notably, these oligomers can be easily accessed *in vitro* under nonphysiological conditions making Prxs unique within the building blocks trialed so far for practical purposes. The use of Prx in bionanotechnology has expanded, and this morpheein has emerged as an adaptable platform where “bottom-up” tailored building of responsive nanostructures and nanodevices can be easily achieved (Figure 1b).

This review collects and discusses the best characterized examples of bioinspired “bottom-up” strategies for the construction of responsive nanostructures and nanodevices using the Prx morpheeins (Figure 1b). The data are presented according to (i) the biochemical and structural features which underlie the morpheein behavior of Prxs, (ii) the chemical and/or genetic engineering expedients applied to stabilize their patterned oligomers, and (iii) the strategies exploited to create nanostructures and nanodevices using the oligomers as templates and scaffolds.

## 2 Typical 2-Cys Peroxiredoxins: Structure–Function Relationship of a Morpheein

Prxs are enzymes with cysteine-dependent peroxidase activity widely found in prokaryotic and eukaryotic organisms.^31^ Their high abundance in cells suggests a remarkable role, a condition common to other ring-shaped proteins formed *in vivo*.^32^ The family of Prxs encompasses six subfamilies grouped according to structural and peptide sequences at the active site containing the reactive cysteine, namely, AhpC/Prx1, Prx6, BCP/PrxQ, Tpx, Prx5, and AhpE, as reported in the peroxiredoxin classification database PREX.^33,34^ The AhpC/Prx1 subfamily is the largest and includes the so-called typical 2-Cys Prxs, a class of proteins with a pair of reactive cysteines forming intersubunit disulfide bonds during the peroxidatic cycle (see subsection 2.1). Some members of this subfamily show the moonlighting behavior as they can act as a peroxidase enzyme to detoxify cells from hydrogen or alkali peroxides as well act as an ATP-independent molecular chaperones (holdases) able to prevent precipitation of unfolded proteins.^33,35,36^ This shift in function is accompanied by a change in shape from homodimers and single rings to high-order oligomers, such as tubes of stacked rings and cage-like particles, due to conformational changes as described at atomic details for the Prx (isoform I) from *Schistosoma mansoni* (*Sm*PrxI)^37,38^ and two mitochondrial Prx (isoforms III) from *Bos taurus* (*Bt*PrxIII, also called SP-22)^30,39^ and *Homo sapiens* (hPrxIII).^29,40^ However, other typical 2-Cys Prxs can form particles and tubes, such as human PrxI and PrxII,^28,41,42^ Thiol Specific Antioxidant I (TsaI) from *Saccharomyces cerevisiae*,^43,44^ and Prx from *Pseudomonas aeruginosa*,^45^ but in these cases, the 3D structures of their high-order oligomers and the molecular mechanism underlying the structural shift have not yet been clarified (see Figure 3). In the next two subsections, the structural–functional switch of *Sm*PrxI will be considered due to its unique morpheein and moonlighting behavior representing the first one investigated by high-resolution X-ray crystallography^37,38^ and exploited in bionanotechnology.^46^ Notably, at least for *Sm*PrxI, the moonlighting behavior strictly depends on the oligomerization state.^2,47^ This gain of function is due to the exposure of hydrophobic surfaces, likely at the rim of the ring, coupled to an increased disorder of its polypeptide making *Sm*PrxI more “sticky” than its orthologues^38^ and thus more prone to potentially interact with different nanomaterials, as similarly observed for other ring-shaped molecular chaperones.^48,49^ Differences in the oligomerization mechanism of human and bovine mitochondrial PrxIII proteins with respect to *Sm*PrxI will be highlighted and discussed.

### 2.1 Low Molecular Weight (LMW) Homo-Oligomers of Typical 2-Cys Peroxiredoxin: Dimers, Rings, and Peroxidase Activity

**Figure 2:**
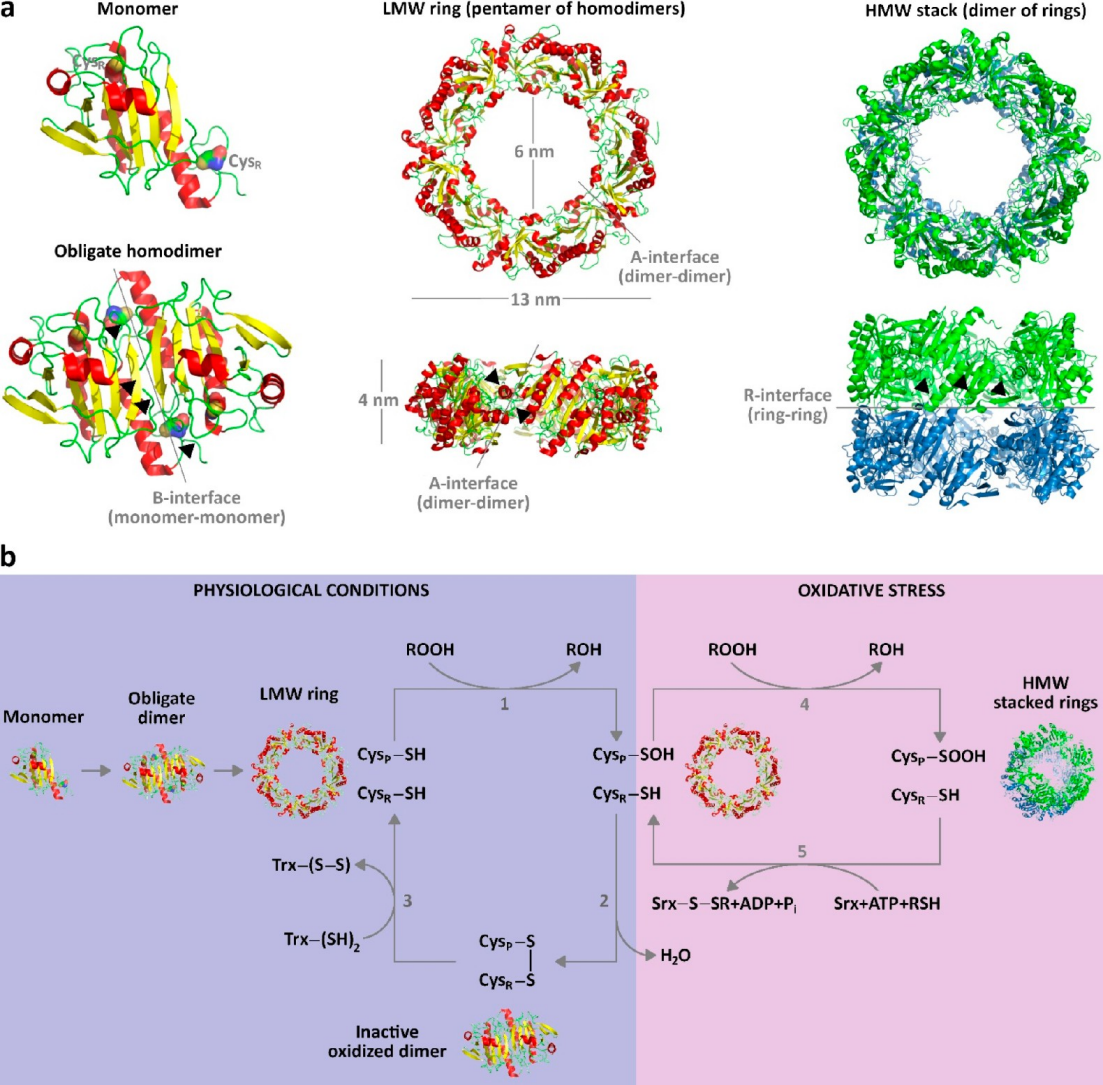
Structure and biochemical activities of typical 2-Cys *Sm*PrxI. (a) Secondary, tertiary, and quaternary structure of *Sm*PrxI. The β-strands and α-helices are in yellow and red cartoons, respectively. The catalytic Cys48_P_ and Cys169_R_ are shown as solvent-accessible surfaces. The elements involved in the monomer–monomer B-interface (β7-strand/antiparallel β7-strand, N-terminal region/β-hairpin/α5-helix, β7-α6 loop/α6-helix, α2-helix/C-terminal region), dimer–dimer A-interface (α4-helix/β3-α2 loop at the external side of the ring, β5-α5 loop/β-hairpin on the internal side), and ring–ring R-interface (α2- and α6-helices of the upper ring/α2- and α6-helices of the lower one, β2-strands of the upper ring/β2-strands of the lower one) are indicated by arrowheads. (b) Step 1: under physiological conditions, the LMW ring peroxidase scavenges peroxides (ROOH) into reduced species (ROH) undergoing oxidation at the catalytic Cys_P_ residues (Cys_P_–SH) into cysteine sulfenic acids (Cys_P_–SOH). Step 2: local unfolding at the active sites and at the C-terminal arms allow Cys_P_–SOH to move toward the resolving Cys_R_ (Cys_R_–SH) forming intersubunit disulfide bridges (−S–S−) which break the ring into oxidized homodimers. Step 3: Trx reduces the disulfide bridges to enable the homodimers to reassemble into a fully folded active ring. Step 4: under oxidative stress conditions, the oxidized Cys_P_–SOH residues undergo further oxidation to form cysteine sulfenic acids (Cys_P_–SO_2_H) triggering structural changes that induce the rings to stack into HMW complexes with holdase and intracellular signaling activity. Step 5: Srx reduces the overoxidized HMW species at the catalytic sites to form Cys_P_–SOH, which, in turn, can undergo further reduction into LMW peroxidase.

Typical 2-Cys Prxs can form supramolecular oligomers made by identical subunits with molecular mass ∼22 kDa and possessing a thioredoxin (Trx)-like fold composed of a seven-stranded β-sheet (β1−β7) wrapped between six α-helices (α1−α6). Within each subunit, the first turn of the α2-helix hosts the so-called peroxidatic Cys_P_, while the flexible C-terminus contains the resolving Cys_R_ involved in the formation of the intersubunit (typical 2-Cys Prxs) or intrasubunit (atypical 2-Cys Prxs) disulfide bonds during the catalytic mechanism (Figure 2a) and absent in 1-Cys Prxs.^50^ Typical 2-Cys Prxs exist as obligate homodimers (∼44 kDa) where the two subunits interact via an isologous interface, called the B-interface. One subunit is rotated 180° with respect to the other one, thus conferring to the homodimer an internal twofold symmetry and placing the catalytic cysteines close to each other, that is, Cys_P_ of one subunit facing Cys_R_ from the other subunit. Therefore, the resulting homodimer contains two identical catalytic sites with twofold symmetry. The B-interface relies on the interaction between (i) the β7-strand from one subunit with the corresponding antiparallel β7-strand belonging to the other subunit resulting in a large 14-stranded β-sheet, (ii) the N-terminal region of one subunit with the β-hairpin and part of the α5-helix of the other subunit on one side of the homodimer, (iii) the β7-α6 loop of one subunit and the α6-helix of the other subunit, and (iv) the α2-helix from one subunit and the C-terminal region belonging to the other one (Figure 2a).^50^

Under reducing physiological conditions, typical 2-Cys Prx homodimers self-assemble into a ring-shaped complex (∼220 kDa), that is usually referred to as low molecular weight (LMW) species. For instance, *Sm*PrxI under reducing conditions at pH = 7.4 is a LMW ring complex made by five obligate homodimers (pentamer of homodimers) resulting in a regular decameric complex (PDB: 3ZTL)^32,37^ and resembling a pentagon with 52-point group symmetry and superimposable with homologous LMW species.^31^ The *Sm*PrxI LMW ring has a thickness of ∼4 nm, a diameter of ∼13 and a pore of ∼6 nm. The seven-stranded β-sheet of each monomer is sandwiched by helices α1 and α5 and one short β-hairpin at the internal face of the ring, and by helices α2, α3, α4, and α6 at the external face. The subunits of each homodimer are correlated by a pseudo twofold axis perpendicular to the plane containing the extended 14-stranded β-sheet that forms an angle of ∼27° with respect to the fivefold symmetry axis of the ring complex. The monomer–monomer B-interface within each homodimer and the dimer–dimer interface formed upon assembly into the LMW ring, so-called A-interface, resemble those observed in other typical 2-Cys Prx rings.^47,51,52^ The dimer–dimer A-interface responsible for the LMW ring assembly is also symmetrical and relies on contacts between (i) the α4-helix of one homodimer and the β3-α2 loop of the adjacent one on the external side of the ring and (ii) the β5-α5 loop of one homodimer and the short β-hairpin of the adjacent one on the internal side (Figure 2a). Similar interactions at the A- and B-interface have also been observed in the LMW species of mitochondrial Prxs, which, however, are dodecameric ring complexes, i.e., hexamers of homodimers, with pore and diameters of ∼7 and ∼15 nm, respectively.^39,40,53^

Typical 2-Cys Prxs alternate between reduced LMW rings and oxidized homodimers as a consequence of the protein’s peroxidase activity and its reduction by cytosolic Trx,^54^ the latter being pivotal in parasites such as *S. mansoni* for fulfillment of redox pathways^55^ and their survival as observed by drug discovery studies.^56−59^ The active sites of the reduced *Sm*PrxI LMW ring are in the so-called Fully Folded (FF) conformation,^50,60^ i.e., residues 47–50 of each subunit, including Cys48_P_, contribute in forming the first turn of the α2-helix, while residues 165–185 of the C-terminal tail, containing Cys169_R_, are folded in a hairpin-like structure. In this conformation, the sulfur atom of each Cys48_P_ is ∼13 Å distant from the sulfur atom of Cys169_R_. This implies that during the catalytic cycle, a remarkable structural rearrangement occurs within each homodimer allowing the formation of both the intersubunit disulfide bridges^50^ and rings to disassemble into homodimers (see below).^34^

Prxs share a similar 2-phase enzymatic mechanism and the same conserved reactive cysteine residue, i.e., Cys_P_, being first involved in the peroxidase reaction. The peroxidase catalytic cycle of the LMW ring starts with the reduction of the peroxide, e.g., H_2_O_2_, formation of sulfenic acid residues (Cys_P_–SOH), and the concomitant release of water. The first step of the reaction requires a thiolate nucleophile to break the O–O bond of the peroxide. In each subunit, Cys_P_ is surrounded by three conserved residues: Pro49, Thr45, and Arg124 (numbering is according to *Sm*PrxI). Pro49 limits the solvent accessibility of Cys48_P_ in its reduced form, while when Cys48_P_ is oxidized, its presence facilitates the α2-helix unfolding allowing interaction with Cys169_R_. Thr45 assists in the binding of H_2_O_2_. Arg124 lowers the p*K*_a_ of Cys_P_ by stabilizing its deprotonated form using the positively charged guanidine group.^61^ In fact, Cys_P_ in typical 2-Cys Prxs has a p*K*_a_ around 6 instead of 8.3, the value of free Cys in solution. The second phase of the peroxidase reaction leads to resolution of the cysteine sulfenic acid residue and distinguishes the three Prx classes.^34^ In the typical 2-Cys Prx class, including *Sm*PrxI and the mitochondrial PrxIII, the α2-helices containing the oxidized the Cys_P_–SOH residues undergo a conformational change from an FF to a Locally Unfolded (LU) state moving apart the pocket formed by the Pro, Thr, and Arg and exposing the oxidized sulfur to the solvent. This conformational change is coupled to a movement of the flexible C-terminal arms of the symmetrical subunit of the homodimer placing the resolving cysteine Cys_R_ close to the sulfenic acid for the formation of an intersubunit disulfide bonds. The LU conformation and the unfolding of the C-terminal arm in each subunit result in weakening of the decameric LMW ring complex until a critical state is reached where it breaks down into oxidized inactive homodimers. However, the cytosolic reductant Trx can process the intersubunit disulfide bridges allowing the oxidized homodimers to self-assemble into the reduced peroxidase LMW ring characterized by the FF conformation at the active site (Figure 2b purple panel).^34^

### 2.2 High Molecular Weight (HMW) Homo-Oligomers of Typical 2-Cys Peroxiredoxin: Tubes of Stacked Rings, Cage-Like Particles, and Molecular Chaperone Activity

The morpheein behavior of typical 2-Cys Prxs allows them to undergo further assembly into high molecular weight (HMW) species where the LMW rings interact to form hollow tubular structures or particles.^35,36,44,62^ For most of the Prx morpheeins, the ability to generate HMW species is correlated with the acquisition of holdase activity and exposure of hydrophobic regions on the ring surface,^38^ while in the case of human mitochondrial PrxIII, the HMW species seems to be correlated with the protection of its peroxidase activity under stress conditions; i.e., stacked rings are still able to reduce peroxides without gaining the holdase activity.^40^ Stacking of rings into HMW tubes occurs upon chemical or physical stimuli such as high concentration of peroxides, pH changes, high temperature, and phosphorylation.^35^ For instance, *Sm*PrxI under acidic conditions at pH 4.2 is an HMW complex of two stacked LMW decameric rings as observed by X-ray crystallography at 3.0 Å resolution (PDB: 3ZVJ).^37^ The interface between the rings is called R-interface and comprises two regions, namely, (i) the helices α2 and α6 of each subunit belonging to the upper ring in contact with the corresponding helices (α2 and α6) of the lower ring and (ii) the β2-strand of each subunit belonging to the upper ring in contact with the corresponding strand (β2) of the lower one (Figure 2a). Upon formation of the HMW complex, all the α2-helices and α6-helices are oriented in such a way that the carbonyl groups, hence the helix dipoles, point toward the R-interface. This creates a negatively charged area centered on the Lys164 and His165 (numbering is according to *Sm*PrxI) located at the end of the α6-helices of both the upper and lower rings. Therefore, the positively charged side chains of Lys and His residues (both are expected to be protonated at pH 4.2) stabilize the negative charge of the helix dipoles, thus contributing to the stabilization of the R-interface.^63^ Because of the 52-point group symmetry, the rings can stack one on top of the other indefinitely, at least in theory. However, in such a complex each ring resembles a “cogwheel” where the secondary structure elements facing the ring–ring R-interface are “pawls” through which two rings are interlocked due to a rotation of ∼10° around the fivefold symmetry axes of a ring with respect to the next one.^32^ It is worth noting that in the crystal structure of the stacked *Sm*PrxI HMW complex the sequence segment 47–49 of each subunit containing Cys48_P_ is disordered with Cys48_P_ being solvent exposed; moreover, the C-terminal tail including Cys169_R_ is unstructured as well.^37^ A similar way of stacking has been found in the 3D structures of the hPrxIII HMW complexes at acidic pH obtained by cryo-electron microscopy (Cryo-EM).^40^ In this case, the dodecamer interlocking is possible by a rotation around the sixfold axis of one ring with respect to the other of ∼8°. The contact at the β2 seems lost in the hPrxIII HMW assembly, but, again, at acidic pH both the first round of α2-helices and the C-terminus tails are unstructured exposing Cys_P_ to the solvent. However, it seems that hPrxIII can stack also at pH 8.5, without requiring the unfolding of the active site, but the resulting stacks are found in the crystal lattice and it is not clear how stable they are in solution.

The protonation of Cys_P_ represents one of the triggers of the morpheein behavior of Prxs, because it disrupts the salt-bridges involving a conserved Arg residue (Arg147 in *Sm*PrxI), known to lower the cysteine’s p*K*_a_, making Cys_P_ a better nucleophile toward peroxides (see subsection 2.1). The positively charged residue comes from a position distant in sequence with respect to Cys_P_ and is kept in place by an interaction with a residue of the second turn of the α2-helix that can be either a Glu, a Gln, or a His in the subfamily members.^64^ The breakage of the salt-bridge between Cys_P_ and R124, induced by acidic pH, unwinds the first turn of α2. This event triggers conformational changes at (i) the B-interface leading to C-terminus unfolding of the parent subunit, (ii) the A-interface, where a slight reorientation of the dimers constituting the ring occurs, and (iii) the R-interface, where the proper alignment of the α2 and α6 due to the resulting dimer orientations allows the proper electrostatic interaction between the same structural elements belonging to another stacking ring. Indeed, the mutation of Cys_P_ to Ser, that mimics cysteine protonation, allows all these Prx to stack constitutively even at physiological pH.^29,38,62^ So, there is a strong coupling between Cys_P_ protonation, active site unfolding, and HMW formation. Another proof about this remarkable linking between the unfolding of the active site and the HMW formation at acidic pH comes from small molecule binding: by means of in-solution studies and crystal structure analyses, it has been demonstrated that sulfate ions bound at the active site force the afore-mentioned Cys48Ser mutant of *Sm*PrxI mutant to adopt a FF conformation bridging Ser48 and Arg147 therefore stabilizing the LMW decameric ring.^38^ Similarly, the effect of sulfate, i.e., the capability to shift from high-order to low-order oligomers, has been observed also in hPrxIII.^29^ Another important trigger for the formation of HMW oligomers is the overoxidation of the Cys_P_ side chain to sulfinic (−SO_2_H) or sulfonic (−SO_3_H) acid, fostered by high peroxide concentrations under oxidative stress conditions. In this case, it is not clear if the structural shift is coupled to active site unfolding, as the crystal structure of the human erythrocyte Prx, where Cys_P_ is in the “sulfinic acid” state, is still characterized by folded α2-helices thanks to a salt-bridge with the conserved Arg;^65^ however, upon overoxidation several Prxs are able to form both particles and small tubes,^36,37^ which are implicated in both cell signaling and holdase activity in intact cells at the expanse of the peroxidatic one (Figure 2b pink panel).^31,35,44,66,67^ However, sulfiredoxin (Srx), a small protein of 14 kDa, specifically reduces the sulfinic form of Cys_P_ restoring the LMW forms and the Prx’s peroxidatic activity.^36^

## 3 Peroxiredoxin-Based Bionanotechnology

A survey of the literature indicates that the structural shift of morpheein oligomers has been best characterized in three typical 2-Cys Prxs, i.e., *Sm*PrxI, hPrxIII, and *Bt*PrxIII. Based on published results, this section is intended to provide information about (i) the genetic engineering/chemical expedients used to stabilize the *Sm*PrxI, hPrxIII, and *Bt*PrxIII oligomers *in vitro*, i.e., rings, tubes, particles, and catenanes and (ii) the strategies utilized to create nanostructures and nanodevices using such oligomers as templates and scaffolds.

### 3.1 Rings, 2D Arrays, Tubes, Particles, and Catenanes: Controlling the Prx Oligomerization

**Figure 3:**
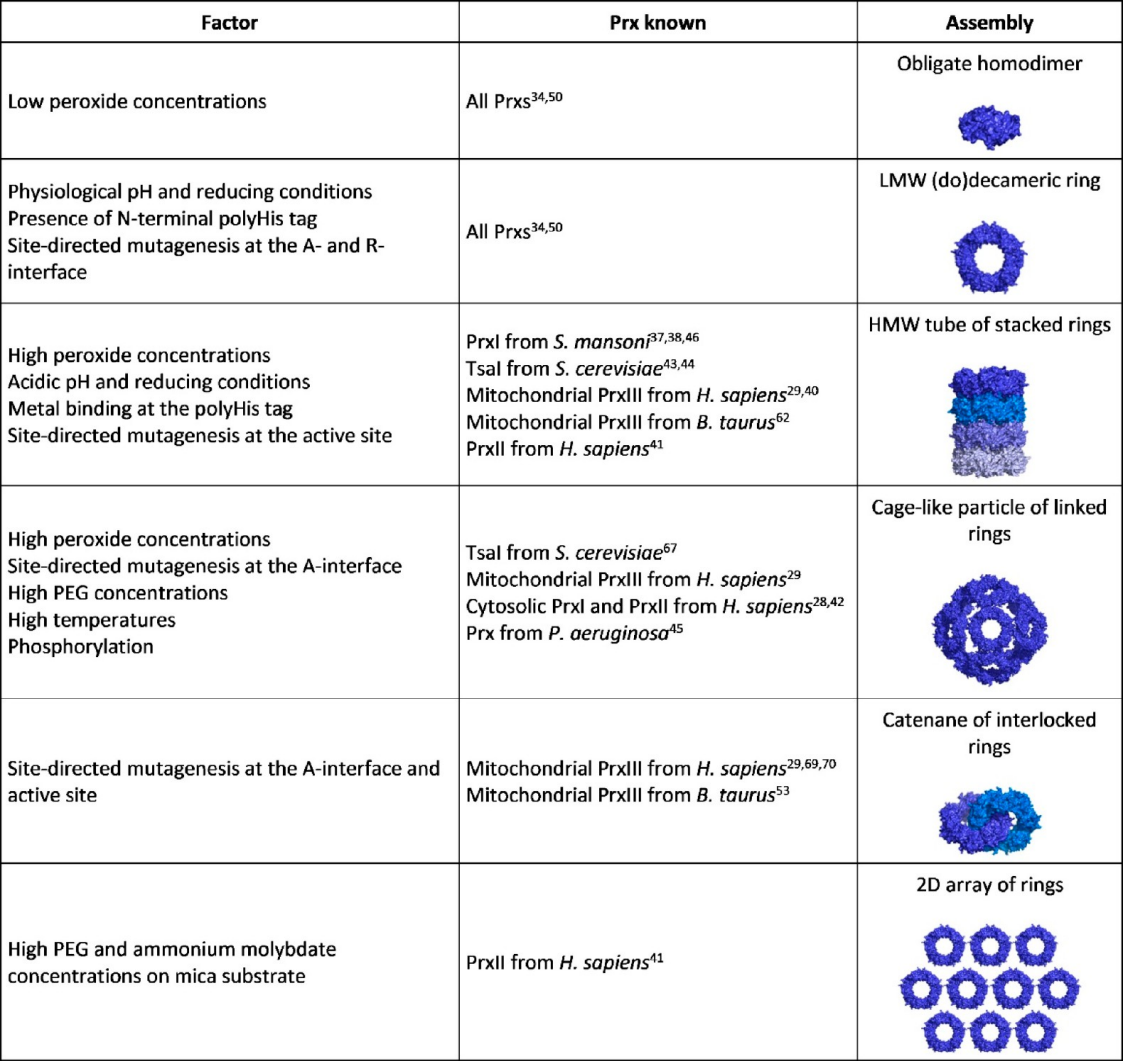
Chemical/genetic engineering factors that master the morpheein behavior of typical 2-Cys Prxs, are reported.

Protein rings, 0D cages, 1D strings, and tubes as well as 2D crystal sheets have generated a great deal of interest in applications including artificial enzyme mimics, light harvesting antenna, drug and imaging nanocarriers, and nanoreactors.^68^ In this view, typical 2-Cys Prx morpheeins show noticeable versatility, as they can naturally self-assemble into rings, tubes, cage-like particles, and catenanes. It is apparent that each Prx assembly described in the following paragraphs depends on specific conformations of the redox-active site lying at the B-interface, which can be induced by particular experimental conditions or site-directed mutagenesis, and on the engineering of the A-, R-interface and of the N-termini (Figure 3).

#### 3.1.1 Prx Rings

Ring proteins are closed oligomers, in which the rotational symmetry between subunits causes saturation of all the subunit–subunit interaction surfaces leading to structures with defined stoichiometry.^32,71^ Therefore, the sites available for binding nanomaterials are periodically and geometrically arranged with nanometric precision on the surface. Ring proteins exhibit four surfaces available for conjugation and for further derivatization: two surfaces above and below the ring plane, the third one around the pore, and the fourth outside with respect to the pore;^32,72^ the bottom and top surfaces are structurally identical as well as the inner and outer ones, as typical 2-Cys Prx rings are made by homodimers with twofold symmetry (see subsection 2.1). These structural properties, the inherent ability to bind nanomaterials and the possibility to engineering the surface (see section 1) make ring proteins ideal tectons in bionanotechnology.^48,72−77^

**Figure 4:**
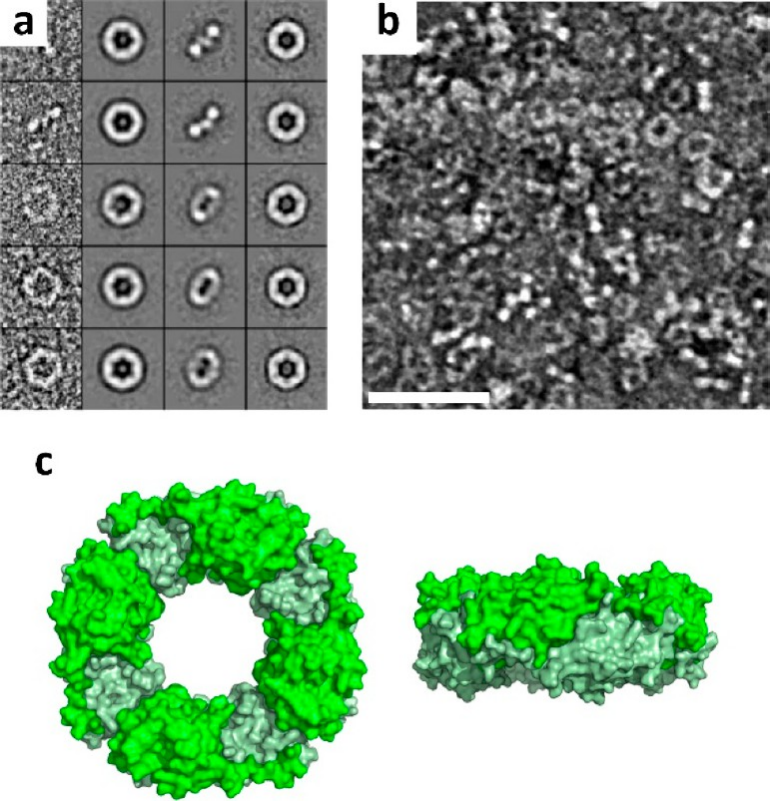
Constrained assembly of Prx rings. (a) hPrxIII rings at pH 8.0 in the presence of reductant TCEP. Adapted from ref (29). Copyright 2014, American Chemical Society. (b) N-terminal polyHis-tagged *Sm*PrxI rings at pH 7.6 (scale bar = 40 nm). Reproduced with permission from ref (46). Copyright The Royal Society of Chemistry. (c) The unusual N-terminal polyHis-tagged *Py*PrxI octameric “miniring” (PDB: 4L0W).

Stable, monodispersed Prxs LMW rings are obtained *in vitro* under reducing conditions at basic pH, i.e., between 7.2 and 8.0. For instance, hPrxIII observed by transmission electron microscopy (TEM) forms rings at pH 8.0 in the presence of reducing agent tris(2-carboxyethyl)phosphine (TCEP). Projection class averages of the rings show the hexagonal dodecameric assembly (hexamers of homodimers) with diameter of ∼16 nm, pore of ∼7 nm, and thickness of ∼4 nm when observed face-on, i.e., with the sixfold symmetry axis perpendicular to the substrate; alternatively, the rings appear as rods when observed side-on (Figure 4a),^29^ in agreement with the crystal structure (PDB: 5JCG).^40^

Alternatively, Prx rings are obtained by genetic engineering at basic pH but without relying on the redox conditions. For instance, hPrxIII engineered with a N-terminal polyHis tag forms rings resembling the untagged protein at pH 8.0,^29^ as confirmed by analytical ultracentrifugation (AUC).^75^ Alike hPrxIII, rings can be obtained using the N-terminal polyHis-tagged *Sm*PrxI at pH 7.6 (Figure 4b)^38,46^ characterized by the pentagonal decameric assembly (pentamer of homodimers; PDB: 3ZTL).^37^ The rings appear as spherical globules with height profile of ∼12 or ∼4 nm when imaged by atomic force microscopy (AFM) and, notably, are stable after heating to 75 °C, therefore demonstrating remarkable thermostability.^46^ An unusual “mini-ring” with square-like octameric assembly (tetramer of homodimers) is obtained using the typical 2-Cys Prx type 1 from *Plasmodium yoelii* (*Py*PrxI) when the first seven N-terminal residues are replaced with the polyHis tag (Figure 4c), as observed by X-ray crystallography (PDB: 4L0W).^78,79^

A different approach relies on *ad hoc* amino acid mutations that prevent stacking of the rings into HMW species by destabilizing the ring–ring R-interface without hampering the ring assembly. For instance, the N-terminal polyHis-tagged mutants His164Glu, His164Ala, and Thr163Val of hPrxIII form rings at pH 7.2 in the presence of the metal chelator ethylenediamine tetraacetic acid (EDTA); conversely, the tagged wild-type protein can still stack,^80^ in contrast to other results (see above).^29^ To explain such unusual behavior, the authors considered the involvement of π–π stacking between the imidazole groups of the polyHis tags from adjoining hPrxIII rings and the possibility that the protein, as an ATP-independent holdase activity, may recognize the 33-amino-acid-long polyHis tag as unfolded peptide thus triggering HMW tube self-assembly, aided by electrostatic interactions between the positively charged tags and the negatively charged α2-helices at the R-interface. EDTA prevents the assembly by sequestering any residual divalent ions present in solution, thus hindering the strong histidine ions coordination bonds between rings which would form HMW complexes (see section 3.1.2). The stabilization of the single rings by point-mutations reflects the importance of the interactions at the R-interface in Prx stacking; the mutation of His164, belonging to the α6-helix of hPrxIII and involved in the HMW assembly^40^ as also observed in *Sm*PrxI,^37^ into the negatively charged Glu or uncharged Ala hinders the ionic and polar contacts necessary to form the HMW stacks (see subsection 2.2). Similarly, mutation of Thr163, also belonging to the α6-helix, into the hydrophobic nonpolar Val prevents the stacking likely abolishing the polar contacts through the hydroxyl group.^81^ Interestingly, oligomers depending on redox conditions are obtained using the untagged hPrxIII carrying the substitution Ser78Ala. In this case, the mutant forms single rings in the presence of TCEP at pH 8.0, while assembling HMW cage-like particles under nonreducing conditions^29^ (see section 3.1.2). Ser78Ala localizes at the A-interface and likely contributes to ring stabilization by increasing hydrophobicity between dimers of hPrxIII. This view is also supported by recent findings on peroxiredoxin from *Aeropyrum pernix,* where cross-linking with small aromatic compounds at residues constituting the A-interface, effectively shifts the dimer-ring equilibrium versus the high order oligomer.^82^

#### 3.1.2 Prx 2D Arrays, Tubes, Particles, and Catenanes

In general, high-order oligomers self-assemble by two main mechanisms: (i) a noncommutative or hierarchical mechanism, where closed structures characterized by point group symmetry self-assemble and must be formed first, thus creating the new surfaces to interact with other identical closed structures leading to the final oligomer; (ii) a commutative mechanism, where single subunits may give rise to infinite polymerization as the interaction surfaces always exposed and free to interact with incoming building blocks. Prx morpheeins employ both mechanisms by changing shape of the single subunits: for instance, Prx HMW cage-like particles and tubes follow a noncommutative mechanism, i.e., LMW rings must form first before assembling into HMW species, while HMW catenanes are likely formed by a commutative mechanism.^32,70^

**Figure 5:**
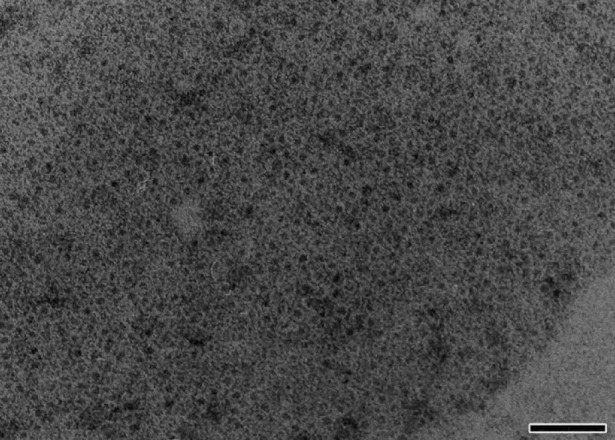
Constrained assembly of 2D Prx ring arrays. Human erythrocyte PrxII adsorbed face-on over mica surface forming large 2D arrays in the presence of PEG and ammonium molybdate (scale bar = 100 nm). Adapted from ref (41), Copyright 2001, with permission from Elsevier.

Complex nanostructures such as 2D crystalline arrays can be obtained using PrxII from human erythrocyte Prx rings adsorbed on flat, unfunctionalized mica surfaces, in the presence of polyethylene glycol (PEG) and ammonium molybdate. Under these conditions, all the rings adsorb face-on over the substrate thus avoiding side-on adsorption that would disrupt the flat 2D arrangement (Figure 5).^41^

**Figure 6:**
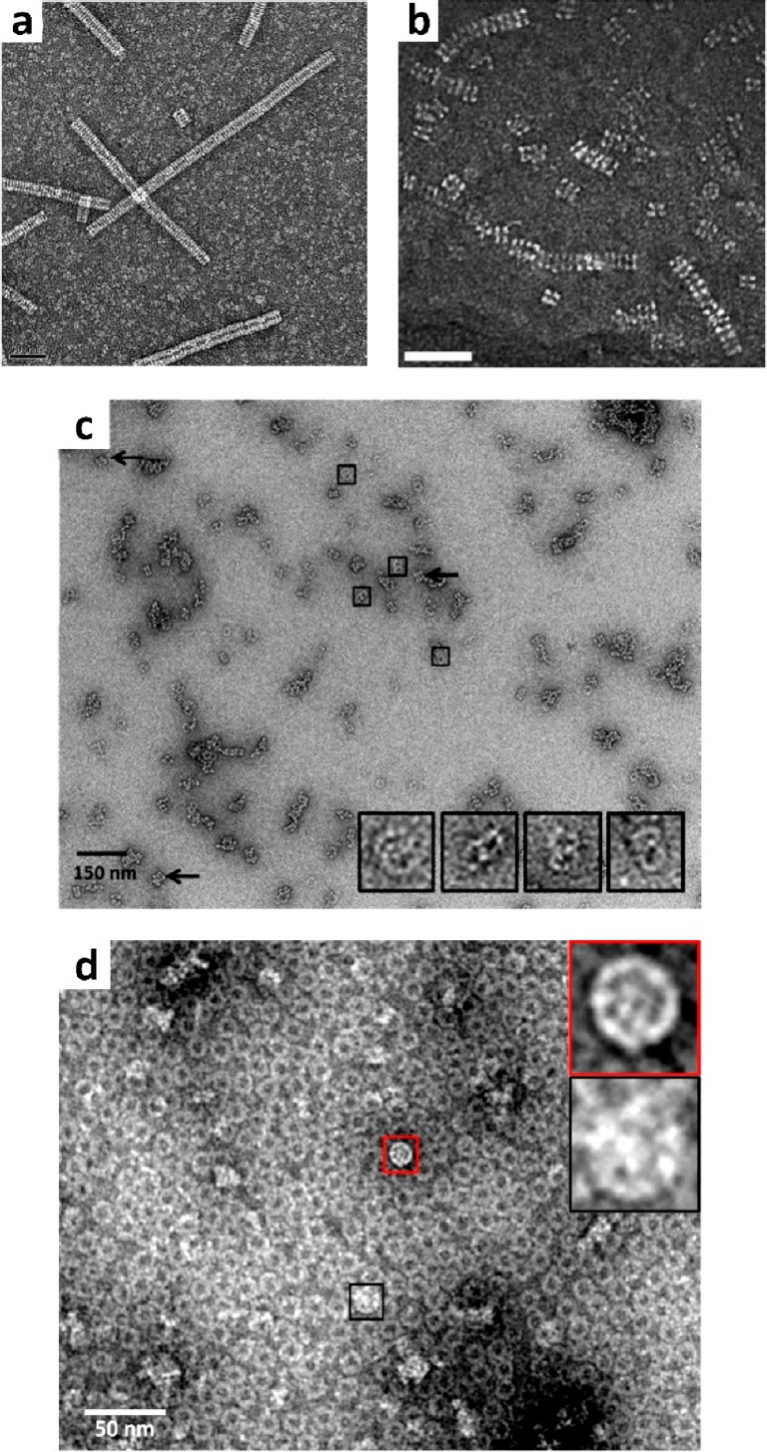
Constrained assembly of Prx tubes, catenanes, and particles. (a) N-Terminal polyHis-tagged hPrxIII tubes at pH 4.0 after tag cleavage (scale bar = 50 nm). Adapted from ref (29). Copyright 2014, American Chemical Society. (b) N-Terminal polyHis-tagged *Sm*PrxI tubes upon imidazole-mediated interaction with Ni^2+^ at pH 7.6 (scale bar = 40 nm). Reproduced with permission from ref (46). Copyright The Royal Society of Chemistry. (c) Catenanes of N-terminal polyHis-tagged Cys47Ser-Ser78Ala double mutant of hPrxIII at pH 7.2. Adapted from ref (29). Copyright 2014, American Chemical Society. (d) Particles assembled at pH 8.0 using the Ser78Ala mutant under nonreducing conditions. Adapted from ref (29). Copyright 2014, American Chemical Society.

Prx can form stable HMW tubes from reversible stacking of rings through pH changes. For instance, the N-terminal polyHis-tagged hPrxIII rings (see section 3.1.1), undergo stacking into long, regular tubes after tag cleavage and decreasing the pH from 8.0 to 4.0 (Figure 6a).^29^ To note, the tubes reversibly and quickly (within tens of seconds) disassemble into rings upon increasing the pH from 4.0 to 8.0 as observed by MS and AUC.^70^ Interestingly, the size of the tubes can be scaled by altering the ionic strength with ammonium sulfate as the average length progressively decreases by adding 100, 200, and 400 mM (NH_4_)_2_SO_4_,^29^ likely due to a sulfate-induced refolding of the active site as observed for *Sm*PrxI (see section 2.2).^38^ These results reflect the physiological behavior of Prx HMW stacks which self-assemble upon exposure to acidic stress and the importance of the interactions established by key residues such as Glu, Lys, and His (Glu20, Lys22, and His164 in hPrxIII) at the α6-helices that stabilize the R-interface between the rings.

The polyHis tags if *ad hoc* engineered can provide an additional site that can be exploited to drive the assembly of tubes. An example is the N-terminal polyHis-tagged *Sm*PrxI ring (see section 3.1.1) that forms hybrid ring-Ni^2+^ complexes able to stack into tubes of 20–90 nm length (Figure 6b). The tubes appear as cylindrical structures with height profile of ∼12 nm and, to note, undergo reversible disassembly upon addition of imidazole likely due to the competition against the polyHis tags for the binding to the metal.^46^ These results account for the high affinity of Ni^2+^ ions for the polyHis sequences (*K*_d_ = 15 nM)^83^ located at the ring pore that become driving forces to achieve stacking into tubes, acting as polymerizing agents increasing the local concentration of the rings. In fact, both the top and bottom surfaces of the polyHis-tagged rings are suitable environments to bind divalent metal ions within a binding surface of ∼130 nm^2^.^46,84^ Similarly, the N-terminal polyHis-tagged hPrxIII constitutively forms short stacks at pH 7.2, even in the presence of EDTA.^80^ Interestingly, changing the length of the tag affects the tube elongation with the number of rings incorporated within the stacks being 2–10, 2–66, and 4–100 for tags containing 2, 4, and 6 histidine residues, respectively.^80^ This effect can be ascribable to two factors modulated by the elongation of the N-termini: (1) π–π stacking of the histidines’ imidazole groups between adjoining rings and/or (2) coordination binding of monovalent and/or divalent metal ions, not efficiently sequestered by EDTA, to the polyHis tags resulting in bridging bonds between rings.

Site-directed mutagenesis of residues belonging to the secondary structure elements involved in the formation of the stacks, as Cys_P_ and Cys_R_, can enable constitutive assembly of tubes without relying on the experimental conditions. Indeed, the mutations Cys48Ser, Cys48Asp, and Cys48Pro of the N-terminal polyHis-tagged *Sm*PrxI and its deletion from residue 166 to 182 at the C-terminal region (ΔC-ter), lead irreversibly to tubes at pH 7.4 whose length depends on the change introduced: the Cys48Asp, Cys48Pro, and ΔC-ter mutants form short stacks of 2 to 6 rings (∼8–24 nm in length), while the Cys48Ser mutant forms tubes composed of 20–30 stacked rings (∼80–120 nm in length).^38^ These results relate with the physiological behavior of *Sm*PrxI as the Cys48Ser and Cys48Asp mutants mimic the Cys–SH and Cys–SO_2_H residues of Cys48_P_ that form upon acid or oxidative stress, respectively, while the Cys48Pro mutant elicits constitutive unfolding of the first turns in the α2-helices favoring the UF conformational state, both required during the formation of HMW stacks; on the other hand, the ΔC-ter mutant removes the steric hindrance caused by the flexible C-terminal tails which hamper the stacking process (see subsection 2.2).^38^ A strong structure–function relationship is thus present in Prxs, as observed for other proteins as well.^85^ Amino acid mutations also allow the assembly of patterned, nontubular Prx HMW oligomers. For instance, complexes of interlocked rings are obtained using the Cys47Ser–Ser78Ala double mutant of the N-terminal polyHis-tagged hPrxIII at pH 7.2 (Figure 6c),^29,69^ resembling catenanes formed by the Cys168Ser and Phe190Leu mutants of *Bt*PrxIII.^53^ These structures seem to rely again on particular structural rearrangements of the Prx’s active site and not on the polyHis tag presence or on experimental conditions. For instance, this HMW state seems to rely on the destabilization of the C-terminus, and thus on conformational changes at the B-interface. The F190L site-directed mutant of *Bt*PrxIII, a residue belonging to the YF motif known to modulate Cys_P_ overoxidation and orientations during the catalytic cycle of typical 2-Cys Prxs, destabilizes the first turns of the α2-helix. The crystal structure of this mutant in the reduced state highlights the presence of an unusual position of the conserved Arg residue, now far from Cys_P_, pointing with its guanidinium group toward the conserved glutamate residue at the second turn of the α2-helix^69^ (see subsection 2.2). Furthermore, the mutation Ser78Ala, located at the dimer–dimer A-interface and far from the R-interface, in the untagged hPrxIII produces HMW cage-like particles of linked rings at pH 8.0 (Figure 6d).^29^

### 3.2 Prx-Based Nanofabrication and Biomaterials

Broadly speaking, when used to control the assembly or synthesis of nanostructures, biomolecules are referred to as “soft templates”. Nanofabrication through the “bottom-up” strategy based on soft templates is efficient to create nanostructures with various morphologies.^86,87^ However, obtaining protein templates with defined, patterned shapes is extremely difficult using computational methods as these macromolecular architectures require complex and elaborate protein–protein interactions and specific control of protein orientation.^88^ This section illustrates how the Prx morpheeins, especially rings and tubes, and Prx-derived peptides can be used to create nanostructures and nanodevices with specific chemical, physical, and biological properties useful for a vast array of applications (Table 1).

**Table 1:** Overall View of All Known Prx-Based Nanostructures and Applications

Prx assembly	Prx known	nanostructure	properties	application^a^
IKHLSVN-derived ribbons	*Bt*PrxIII	Nanoribbons^89^	Colloidal	Hydrogel^89^
			Self-assembling	
	(SP-22)	Nanofiber-like optoelectronic compounds^90^	Colloidal	1D conductive nanofilaments in field-effect transistors^90^
			Self-assembling	
			Semiconductive	
LMW (do)decameric rings	*Sm*PrxI	Ring-trapped gold NPs^46^	Colloidal	Nanoelectrodes
			Self-assembling	
			Electrically responsive	
		Ring-shaped gold NPs arrays^84^	Colloidal	SERS nanoprobes
		GO composites doped with ring-trapped metal NPs^77,91^	Colloidal	3D metal-doped graphene nanoscaffolds for gas sensing
			Self-assembling	
			Low weight	
			Microporous	
		Ring-shaped silver rings on graphene-coated solid-state membranes^92^	Colloidal	Nanopores for single-molecule detection and sequencing
			Self-assembling	
			Plasmonic	
			Fluorescent	
		Ring-linked gold NPs arrays^84^	Colloidal	Nanometric probes for intracellular Raman imaging
			Self-assembling	
			Plasmonic	
			SERS	
		Ring-doped GO composites^93^	Biocompatibile	Scaffolds for cell differentiation and growth^93^
			Pro-differentiating	
	hPrxIII	Ring-trapped iron NPs arrays^94^	Colloidal	Nanoelectrodes
			Electrically responsive	
		Ring-tethered gold layers^75^	Preferential ring orientation	Biosensors
		Ring-tethered gold layers coated with SAM^75^	Preferential ring orientation	Biosensors
HMW tubes of stacked rings	hPrxIII	Tube-trapped iron NPs arrays^94^	Colloidal	1D conductive nanofilaments
			Self-assembling	
			Electrically responsive	
		Tube-tethered gold layers^75^	Preferential tube orientation	Biosensors
		Tube-tethered gold layers coated with SAM^75^	Preferential tube orientation	Biosensors
	*Sm*PrxI	Tube-trapped gold NPs arrays^46^	Colloidal	1D conductive nanofilaments
			Self-assembling	
			Electrically responsive	
		Tube-containing coatings^95^	Biocompatibile	Scaffolds for cell differentiation and growth^95^
			Pro-differentiating	
	*Bt*PrxIII	Tube-containing coatings^95^	Biocompatibile	Scaffolds for cell differentiation and growth^95^
	(SP-22)		Pro-differentiating	

a It includes all known (referenced) and putative potential applications (unreferenced).

#### 3.2.1 3D Hydrogels and Bio-Organic Field-Effect Transistors

**Figure 7:**
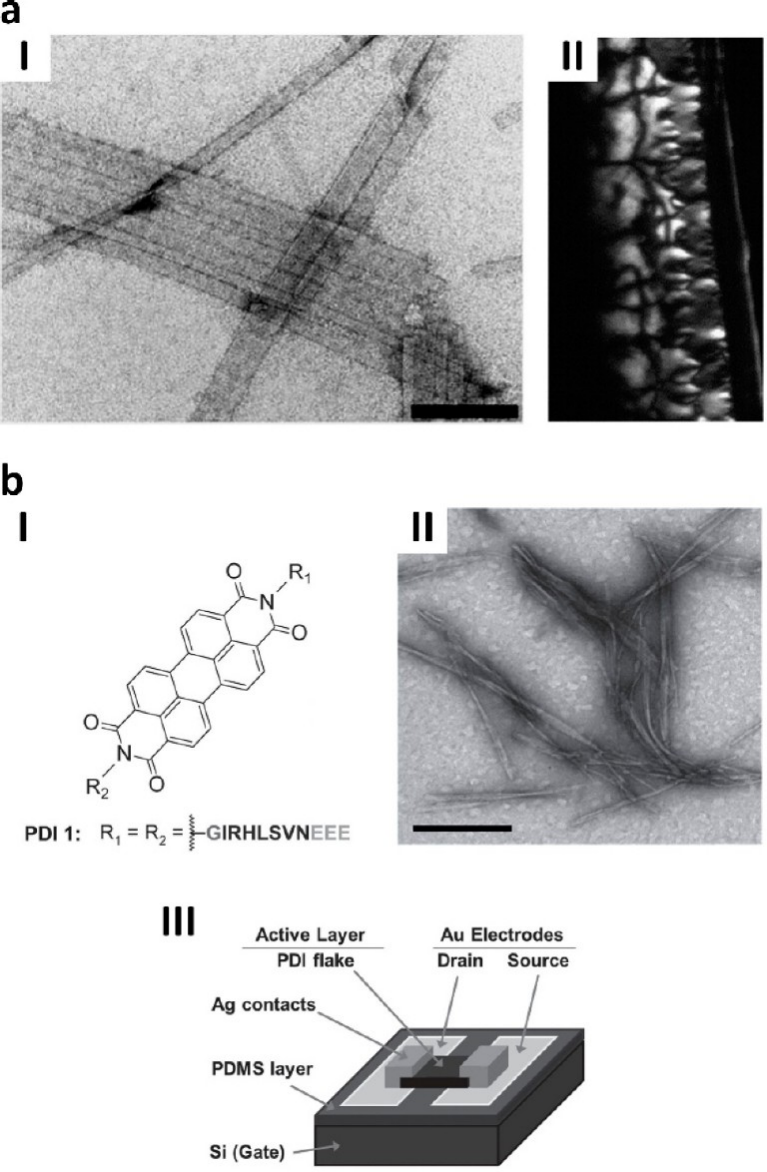
Prx peptide-based hydrogels and semiconductive nanofibers for biOFET. (a) Nanoribbons (I) and hydrogel (II) obtained by the self-assembling *Bt*PrxIII-derived IKHLSVN peptide (scale bar = 200 nm). Adapted from ref (89). Published by The Royal Society of Chemistry. (b) Hybrid compound obtained by conjugation between the GIRHLSVNEEE derivative and PDI (I) and its self-assembly into elongated nanofibers (II, scale bar = 200 nm) that are utilized as semiconductive layers between Ag contacts in biOFET (III). Adapted with permission from ref (90). Copyright 2015 WILEY-VCH Verlag GmbH & Co.

The B-interface in typical 2-Cys Prx homodimers relies on contacts between the seven-stranded β-sheet from one monomer and the same secondary structure of the other monomer, resulting in an extended 14-stranded β-sheet (see subsection 2.1). It is observed that this structure is found in about 15% of native homodimer interfaces of different Prx families and frequently includes the amino acidic sequence Ile-Lys-His-Leu-Ser-Val-Asn (IKHLSVN).^89^ The IKHLSVN sequence derived from *Bt*PrxIII can be synthesized as a water-soluble, stand-alone peptide with both N-acetylated and C-amidated regions to maintain the net charge of the corresponding interface. This peptide forms 3D birefringent hydrogels at concentrations between 20 and 200 mg mL^–1^ in pure water exhibiting liquid crystalline texture and resulting from spontaneous self-assembly of elongated nanoribbons (Figure 7a).^89^

The IKHLSVN peptide can be employed to obtain perylene imide-based organic semiconductor assemblies for transistor devices. Namely, the peptide derivatives glycine-IRHLSVN-glutamate-glutamate-glutamate (GIRHLSVNEEE) and N-acetylated EEEIRHLSVN-ethylamine (Ac-EEEIRHLSVN-ethylamine) can be conjugated with perylene diimide (PDI) or perylene imide bisesters (PIBEs) to obtain hybrid bio-organic compounds with luminescence efficiency and optoelectronic properties. While the glycine and ethylamine groups act as low-steric linkers between the peptides and the organic compounds, the three glutamate ionizable residues improve the solubility of the final hybrids and provide a means to invoke pH-triggered self-assembly. The compounds form diffusive, interlaced networks of nanofibers with roughly 200–300 nm up to micrometer length, likely due to the synergistic effect of the IRHLSVN self-assembly behavior and the π–π stacking between PDI or PIBEs. By taking advantage of the nanofibers as semiconductor layers between Ag electrodes, bio-organic field-effect transistors (biOFET) can be built showing output and transfer characteristics upon exposure to voltages (Figure 7b).^90^

#### 3.2.2 Colloidal 0D NPs

**Figure 8:**
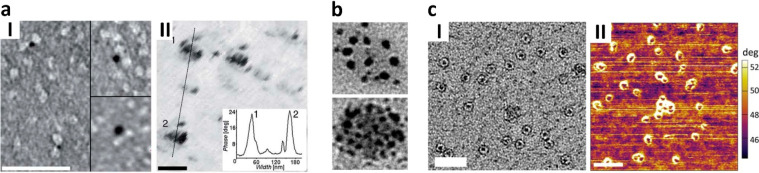
Colloidal Prx-embedded NPs. (a) Ni^2+^-coated 1.8 nm AuNPs trapped within N-terminal polyHis-tagged *Sm*PrxI rings (I, scale bar = 40 nm) and their electrical response observed by EFM upon application of a voltage of 6 V on silicon dioxide (II, scale bar = 40 nm). Reproduced with permission from ref (46). Copyright The Royal Society of Chemistry. (b) Circular arrangement of bare 2 nm AuNPs adsorbed over *Sm*PrxI rings. Adapted from ref (84), Copyright 2020, with permission from Elsevier. (c) Templated synthesis of FeNPs within N-terminal polyHis-tagged hPrxIII rings (I, scale bar = 50 nm) and their EFM electrical response on mica under 5 V bias voltage (II, scale bar = 100 nm). Adapted from ref (94). Copyright 2018, American Chemical Society.

The inborn affinity of amino acids toward metals^4,8^ and the possibility to insert polyHis sequences to further expand this affinity allow Prx to bind metals and build hybrid Prx-metal nanostructures.^9,83,96^ For instance, imidazole-mediated binding of Ni^2+^-coated 1.8 nm AuNPs to N-terminal polyHis-tagged *Sm*PrxI rings can be achieved at pH 7.6. The nanoparticles are partially trapped within the ring scaffolds thus giving to the hybrid *Sm*PrxI-AuNPs complexes electrical responsiveness if exposed to a 6 V bias voltage, as demonstrated by repulsive-mode electrostatic force microscopy (EFM) performed in air on samples adsorbed on silicon dioxide substrates (Figure 8a).^46^ Furthermore, the binding affinity for gold of the ring without dependence on the polyHis tag is observed using bare 2 nm AuNPs. In this case, the nanoparticles appear adsorbed all over the ring surface yielding arrays with circular arrangement and size of ∼21.2 nm (Figure 8b),^84^ thus fitting with size scale of the crystal structure (PDB: 3ZTL).^37^ On the other hand, templated synthesis of nanoparticles can be obtained taking advantage of the polyHis tags as nucleation sites. For instance, binding of Fe^2+^ ions to N-terminal polyHis tagged hPrxIII rings is achieved by addition of Fe^2+^ at pH 8.0 and 4 °C in the presence of citrate, which stabilizes the ions and prevents oxidation by molecular oxygen. Under these conditions, the Fe^2+^ ions are captured in the ring pore to allow seeding growth of electron-dense ∼4 nm iron oxyhydroxide NPs (FeNPs), which confer electrical responsiveness to the complex adsorbed on mica substrates upon application of a 5 V bias voltage in air (Figure 8c).^94^

These examples show how to generate stable, responsive metal–protein complexes by tidily regulating the strong metal–ligand coordination bonds and the weak ones occurring in the protein quaternary assembly to avoid precipitation as well as disassembly of the complex. For instance, imidazole used for assembling the *Sm*PrxI-AuNP complexes is efficient in balancing the strong nickel-polyHis interaction (*K*_d_ = 15 nM)^83^ and the weaker molecular contacts holding the *Sm*PrxI LMW ring (*K*_d_ ≈ 1 μM for the dimer/decamer equilibrium).^38,97^ Moreover, hybrid protein–NP conjugates represent tools for addressing many of the difficulties in biomedicine, e.g., as biocompatible drug delivery systems: these hybrid nanostructures, indeed, improve interactions with biological material such as cells because the protein coating can increase penetration of cell membranes by NPs.^98^

#### 3.2.3 NPs-Graphene 3d Composites and 2d Plasmonic Nanopores Arrays

**Figure 9:**
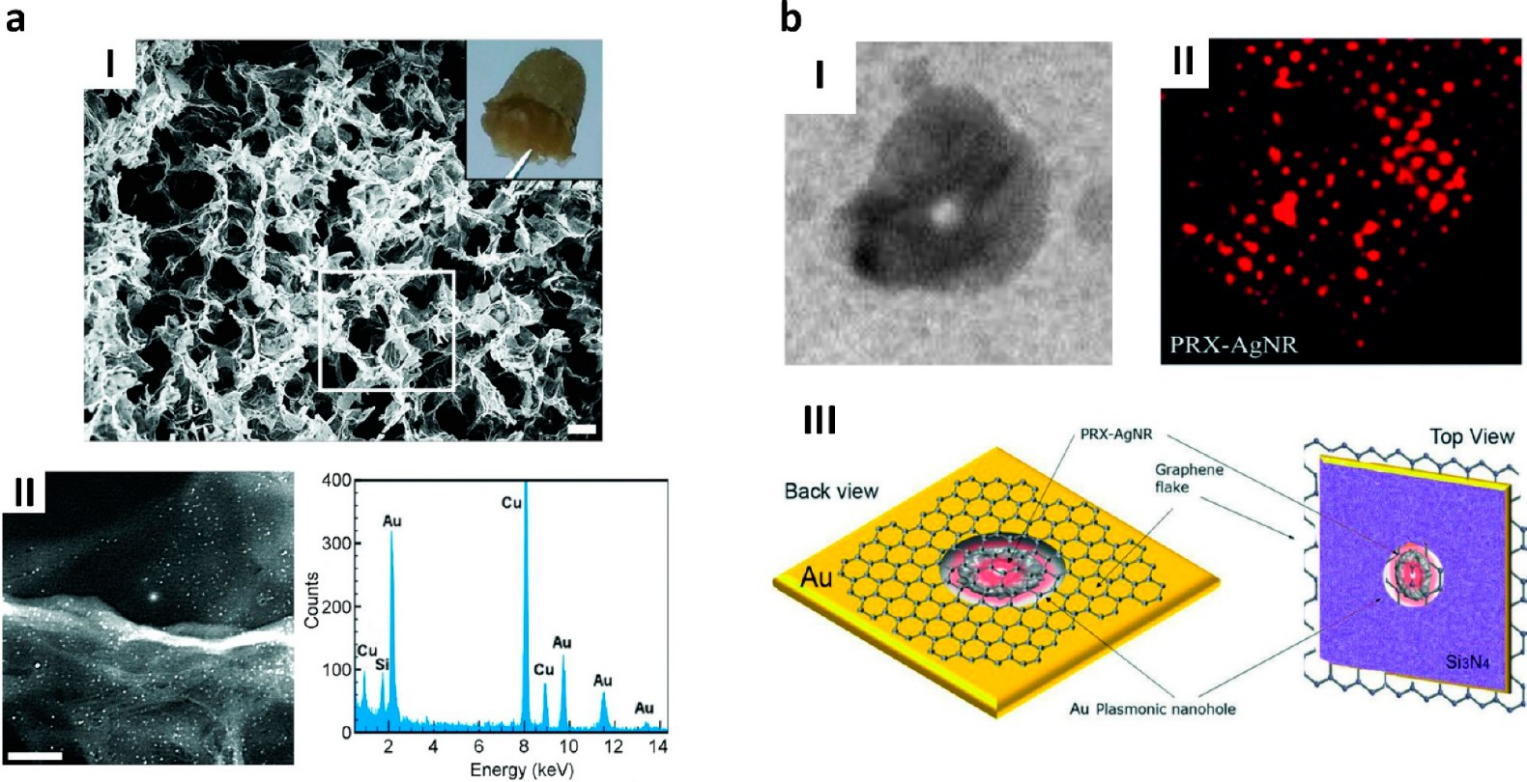
Prx-templated NPs-rGO composites and plasmonic nanopores arrays. (a) Micrographs of 3D microporous rGO obtained upon interaction with N-terminal polyHis-tagged *Sm*PrxI rings (I, scale bar = 10 μm). The inset shows a picture of the material obtained after freeze-drying. The composite can be doped with AuNPs using the *Sm*PrxI scaffold as linker and assessed by EDS elemental analysis reveling the presence of gold (II, scale bar = 50 nm). Reproduced with permission from ref (77). Copyright The Royal Society of Chemistry. (b) Ag nanorings synthesized on *Sm*PrxI ring templates (I) and adsorbed on graphene before labeling with fluorophores and deposition on solid-state 2D nanopores arrays (II). Structure of the final hybrid nanodevice with plasmonic behavior at the nanoring pore (III). Adapted with permission from ref (92). Copyright 2019 WILEY-VCH Verlag GmbH & Co.

Graphene-based materials can be obtained taking advantage of the inherent affinity of proteins toward the 2D lattice of graphene.^6^ As an example, the N-terminal polyHis-tagged *Sm*PrxI rings can be utilized to drive aggregation of 0.3–1 μm GO layers into a microporous 3D composite at pH 7.4, which can be freeze-dried and observed by scanning electron microscopy (SEM). Furthermore, scanning TEM (STEM) and energy dispersive X-ray spectrometry (EDS) demonstrate that the rings can act as scaffolds or templates to dope the composite with Ni^2+^-coated 1.8 AuNPs or ∼3.3 nm palladium NPs (PdNPs) obtained by chemical reduction of Pd^2+^ (Figure 9a).^77,91^ To note, the rings adsorb face-on over the lattice and perform chemical reduction of GO into rGO at the expanse of the native cysteine residues.^77,91^ This example demonstrates how to master the assembly of complex materials by exploiting the biochemical and structural features of the Prx morpheeins: stacking is achieved taking advantage of the perfect symmetry of *Sm*PrxI and its identical top and bottom surfaces (see section 2.1) as well as the protein-catalyzed reduction into rGO, which is known to yield of 3D graphene-based materials.^99^

The graphene-bound Prx rings can be exploited to precisely place metal nanorings on regular, porous 2D arrays in order to assemble devices with improved properties. For instance, N-terminal polyHis-tagged *Sm*PrxI rings can be used as templates to synthesize Ag nanorings with diameter and pore of ∼28 and ∼3 nm, respectively, on graphene layers by chemical reduction of Ag^+^. The nanorings can be labeled with fluorescent dyes and selectively placed on 2D nanopores arrays on solid-state silicon nitride (Si_3_N_4_) membranes as observed by confocal fluorescence microscopy (CM) (Figure 9b),^92^ taking advantage of electrophoresis as reported for other 2D lattices.^100^ The hybrid nanopores show improved fluorescence lifetimes of 1.0 ± 0.8 ns, corresponding to half of the values obtained using silver-free labeled *Sm*Prx1 rings thus suggesting plasmonic behavior of the device. Notably, drilling of 2 nm holes on the graphene surface at the nanoring pore can be achieved by focused electron beam resulting.^92^ This strategy accounts on a synthesis at pH 5.5 in citrate buffer that stabilizes the LMW ring oligomer and acts as adjuvant for Ag^+^ binding by providing a net negative charge to the citrate-coated protein. In addition, at pH 5.5 the polyHis sequences are mostly protonated (p*K*_a_ of histidine is 6) thus discouraging their binding to Ag^+^ to the advantage of the native surface amino acids of *Sm*PrxI.^101^

Self-assembly of graphene-based materials represents a feasible route to tailor and enhance the properties of graphene while making more manageable 3D structures and devices for practical applications, e.g., energy conversion/storage and environmental decontamination.^102^ For instance, the device reported herein represents one of the first examples of hybrid plasmonic nanopores integrated on solid-state membranes that would be useful for next-generation sequencing and single-molecule detection.^103,104^

#### 3.2.4 Gold-Tethered 2D Protein Rings and Colloidal Plasmonic 1D AuNPs Arrays

**Figure 10:**
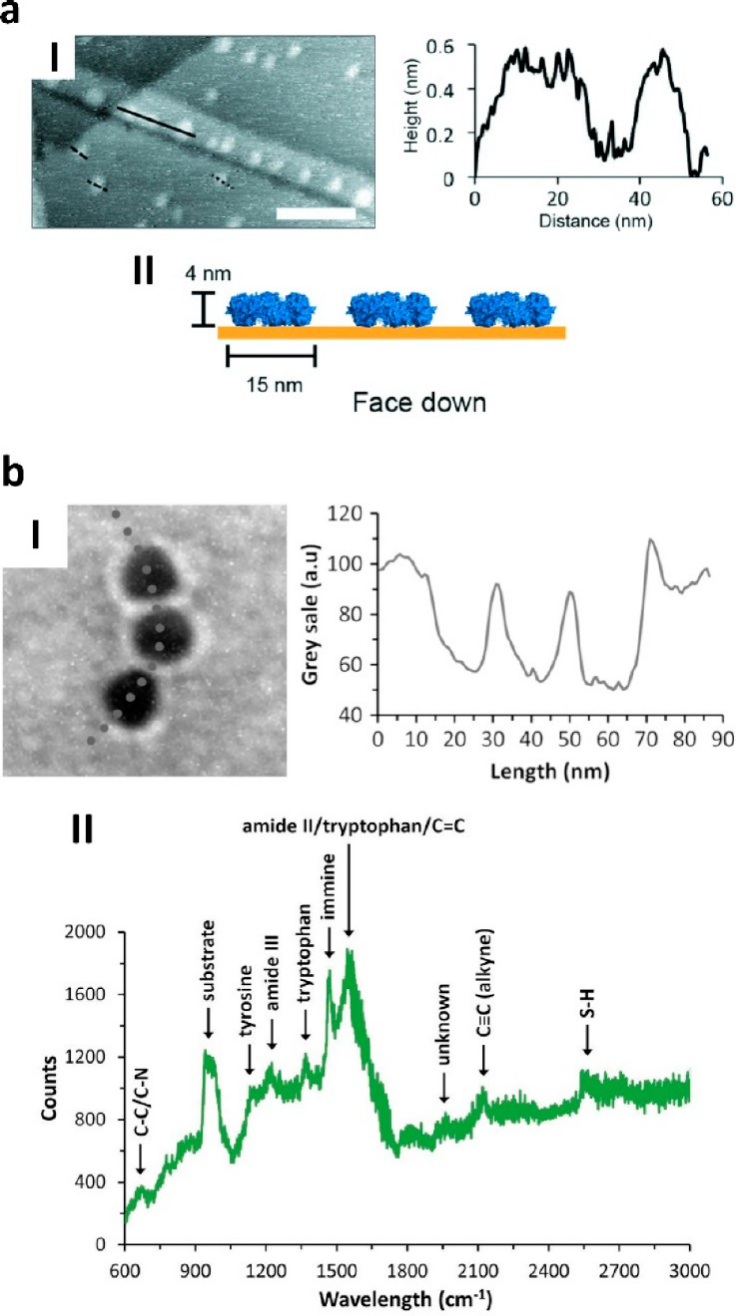
Gold-tethered Prx rings and plasmonic AuNPs arrays. (a) 2D flat assemblies of N-terminal polyHis-tagged hPrxIII rings on gold layers (I, scale bar = 50 nm) adsorbed with preferential face-on orientation (II). Adapted from ref (75), with the permission of AIP Publishing. (b) 1D alkyne-labeled AuNPs arrays assembled upon interaction with N-terminal polyHis-tagged *Sm*PrxI rings after negative staining (I). The arrays show plasmonic effects due to Raman scattering from both amino acids and alkyne tags (II). Adapted from ref (84), Copyright 2020, with permission from Elsevier.

Gold surfaces are useful platforms to create protein-reactive assemblies that can be exploited to build nanodevices for bioelectronic and biosensing applications.^105^ An example of Prxs assemblies on flat Au is the N-terminal polyHis-tagged hPrxIII rings that adsorb at low concentration of 5 μg mL^–1^ and pH 7.2 over mica substrates coated with 300 nm Au layer. Scanning tunneling microscopy (STM) show that that the rings preferentially bind face-on to the gold appearing as discrete globules with 15 nm diameter and 0.5 nm thickness, thus creating a flat protein coating (Figure 10a).^75^ To note, the stability of the interaction is increased by treating the gold surface with a self-assembling monolayer (SAM) of SH-nitrilotriacetic acid (NTA-thiol).^75^

These data represent the first example of building Prx supramolecular structures with preferential orientation tethered to gold surfaces, which would be particularly useful in the preparation of ordered functional 2D or 3D nanoscale assemblies for different applications, including cell, virus, and bacterial adhesion, as well as biomaterial and biodevice engineering.^105^

The affinity of Prxs toward gold can be exploited to assemble colloidal 1D metal NPs arrays labeled with molecular tags, which can find use in applications based on surface enhanced raman scattering (SERS). Namely, 20 nm AuNPs can be labeled with alkyne-based compounds through sulfur–gold interactions, as previously reported for silver nanostructures,^106,107^ and induced to self-assemble upon binding to N-terminal polyHis-tagged *Sm*PrxI rings. The hybrid alkyne-labeled *Sm*PrxI-AuNP complexes are arranged as short 1D arrays with the interparticle gaps indicating the position of the *Sm*PrxI rings. Notably, the arrays exhibit Raman scattering enabling detection of the amino acids and alkyne molecules, the latter being distinguishable for their C≡C bonds in a biomolecule-silent spectral region (Figure 10b).^84^ Alike the *Sm*PrxI-GO hybrid composites where the patterned ring shape drives stacking of GO (see section 3.2.3), the 1D assembly of AuNPs is likely to occur due to the geometrical protein complex and the interaction with the polyHis-tagged identical bottom and top surfaces;^9,96^ moreover, even the native amino acids, including methionine and cysteine, at the *Sm*PrxI ring surface would play a role in the assembly process for being sensitive to changes of pH and ionic strength.^8,84^

By comparison, 1D assemblies of nanoparticles have not been as thoroughly explored as their 2D and 3D counterparts because of the difficulties in their preparation and isolation for analysis. However, 1D assemblies have potential applications in a variety of optoelectronic, electronic, photonic, and magnetic purposes.^108^ For instance, these nanostructures are currently exploited for nondestructive intracellular imaging by Raman spectroscopy, where they are used as nanoprobes in theranostics, e.g., controlled NPs delivery and imaging^107,109^ and plasmonic photothermal treatment.^68^

#### 3.2.5 Graphene-Based 3D Scaffolds for Cell Growth and Differentiation

**Figure 11:**
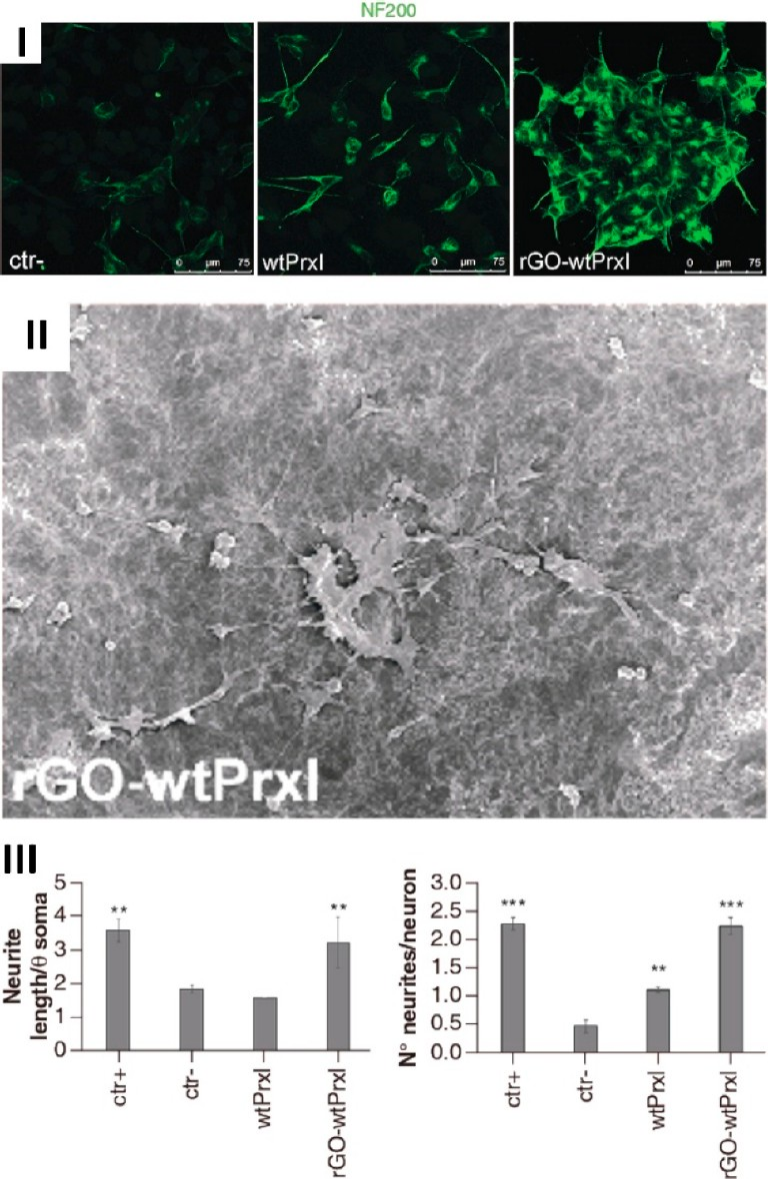
Prx-conjugated rGO scaffold for tissue engineering. SH-SY5Y cells grown on *Sm*PrxI-conjugated GO differentiate with a neuronal-like phenotype and show increased expression of the neuronal marker NF200 as demonstrated by IFM; this effect is much less evident in control cells grown on bare GO or *Sm*PrxI alone (I). The neural-like phenotype is highlighted by electron micrographs showing a network of cells with sprouting long neurites (II) and confirmed by measuring the number and length of the neurites; to note, cells differentiated on *Sm*PrxI-conjugated GO are comparable to positive control samples differentiated with addition of N2 (III). Adapted from ref (93) with permission of Future Medicine Ltd.

As 3D graphene-based composites doped with inorganic nanomaterials are being exploited for electronic and environmental purposes,^102^ protein-graphene materials show interesting properties in biological applications, taking advantage of the inherent biocompatibility of GO.^110^ For instance, polyHis-tagged *Sm*PrxI rings adsorbed on GO can be used as substate for adhesion, growth, and differentiation of human neuroblastoma cells SH-SY5Y into neuronal-like cells without relying on the pro-differentiating agent N2 as observed by immunofluorescence microscopy (IFM). The effect fits with the concurrent expression of the marker of neuronal NF200 and leads to morphological changes of the neuronal-like phenotype and formation of a cell network over the hybrid *Sm*PrxI-GO substrate as confirmed by an increasing neurite length and neurite/neuron number (Figure 11).^93^ Furthermore, the YAP and TAZ transcriptional coregulators are found to move across the cell, from nucleus to cytoplasm, likely acting as molecular triggers for mechano-transduction process of differentiation.^111^ Accordingly, down-regulation of proliferating and pluripotency factors such as RhoA and Sox2 is observed in cells cultured over the *Sm*PrxI-rGO composite.^93^ The differentiation is attributed to increased stiffness gained by GO^112^ likely due to *Sm*PrxI-induced reduction to rGO (see section 3.2.3).

Graphene-based scaffolds for tissue engineering are now at the forefront in medicine as attractive materials for their biocompatibility, versatile chemical states, suitable flexibility, and physicochemical properties, the latter necessary for stabilizing the growth and differentiation of cells. In this context, neural cells are currently considered as the main model for tissue engineering to be assessed in regenerative therapies for various diseases and disorders, e.g., spinal cord injuries, strokes, and Alzheimer’s as well as Parkinson’s disease.^113^ However, even though the surface chemistry of graphene is known to play a significant role in influencing the cell behavior and fate, there is little known about molecular mechanisms underlying the differentiation by mechanical stimuli of the intracellular response and, therefore, this topic remains a hot issue to be fully explored.

#### 3.2.6 Colloidal 1D Polarizable NPs Arrays

**Figure 12:**
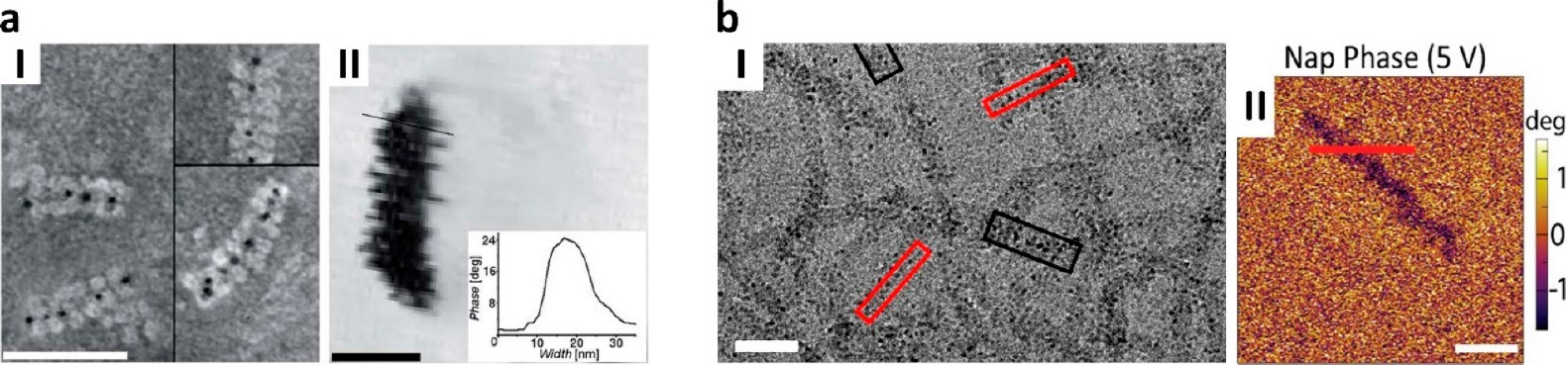
Polarizable Prx-embedded NPs arrays. (a) 1D arrays of Ni^2+^-coated 1.8 nm AuNPs trapped within N-terminal polyHis-tagged *Sm*PrxI tubes (I, scale bar = 40 nm) and their electrical response upon application of a voltage of 6 V on silicon dioxide (II, scale bar = 40 nm). Reproduced with permission from ref (46). Copyright The Royal Society of Chemistry. (b) 1D arrays of FeNPS synthesized on N-terminal polyHis-tagged hPrxIII tubes induced at pH 6.0 (I, scale bar = 50 nm) and their electrical response under 5 V on mica (II, scale bar = 60 nm). Adapted from ref (94). Copyright 2018, American Chemical Society.

Nanometric assemblies with 1D architecture or nanotubes are popular structures and probably the best characterized. In this regard, protein tubes of self-assembling Prx rings can be obtained with special features for wide applications. For instance, N-terminal polyHis-tagged *Sm*PrxI rings can bind Ni^2+^-coated 1.8 nm AuNPs, taking advantage of the Ni^2+^-polyHis interaction, slowly undergoing imidazole-mediated self-assembly into *Sm*PrxI-AuNPs hybrid 1D arrays where the nanoparticles locate between adjoining rings, thus acting as gold metal linkers between adjacent protein molecules. The arrays appear as ∼12 nm cylinders and exhibit EFM electrical responsiveness along the whole structure if adsorbed on silicon dioxide and exposed to a 6 V bias voltage, likely due to the presence metal embedded within the tube cavity (Figure 12a).^46^ Though the polyHis tag-Ni^2+^ interaction is likely the main driving force of the assembly process (see section 3.2.2), even the ring–ring R-interface can play a role in the ring stacking as AuNPs are small enough to partially penetrate the ∼6 nm ring pore; thus, the nanoparticles act as driving forces to increase the local protein concentration thus inducing the conformational changes required to stabilize the electrostatic and polar contacts at the R-interface (see sections 2.1 and 2.2).

As an alternative strategy to achieve 1D assemblies, metal NPs can be obtained by direct chemical synthesis on the Prx ring scaffold and triggering of the stacking through pH changes. For instance, the N-terminal polyHis-tagged hPrxIII rings can be used as templates at pH 8.0 to synthesis at the ring pore ∼4 nm FeNPs O_2_-mediated to oxidation of citrate-stabilized Fe^2+^ ions (see section 3.2.2). Taking advantage of the pH-sensitivity of hPrxIII (see section 3.1.2), on decreasing the pH to 6.0, the hPrxIII-FeNPs complexes can be induced to stack, forming long regular 1D arrays that, according to their hybrid composition, exhibit electrical response along the whole structure when applying voltages of 5 V when adsorbed on mica (Figure 12b).^94^ To note, oxyhydroxide NPs forming the 1D arrays appear embedded within the cavity but also lying at the outer surface of the hPrxIII stacks likely due to partial deprotonation of histidines of the polyHis sequence at pH 6.0, with loss of affinity for the mineralized iron.^94^ In this process, the stacking is likely due to the protonation of the catalytic Cys_P_ (Cys47_P_ in hPrxIII) inducing the conformational changes necessary to stabilize the ring–ring R-interface as reported.^29^

Nanotubes are found widely as structures made by carbon, metals, silicon, and DNA as well as peptides and proteins, the latter occurring naturally in cells as self-assembling structures, e.g., microtubules^16^ and the bacterial flagella.^114^ Due to their highly desirable properties such as biodegradability, biocompatibility, and ease of tailored surface functionalization, protein nanotubes currently found several applications in food, pharmaceutical, and cosmetics sectors. These reasons make them a promising alternative to carbon nanotubes, which, even though possessing excellent tensile strength, large surface area and appropriate electronic, thermal and chemical properties, still evoke concerns about their toxicity and compatibility with biological matter.^115,116^ Though small nanoparticles do not exhibit coupling phenomena, such as surface plasmonic resonance or scattering to be used for instance as biosensors, their regular arrangement into 1D arrays confer the final nanostructure with other properties useful in applications such as conductive nanowires^117^ and nanostructured catalysts for oxygen reduction reaction in fuel cells.^118^

#### 3.2.7 Gold-Tethered 2D Protein Tubes and Scaffolds for Cell Growth and Differentiation

**Figure 13:**
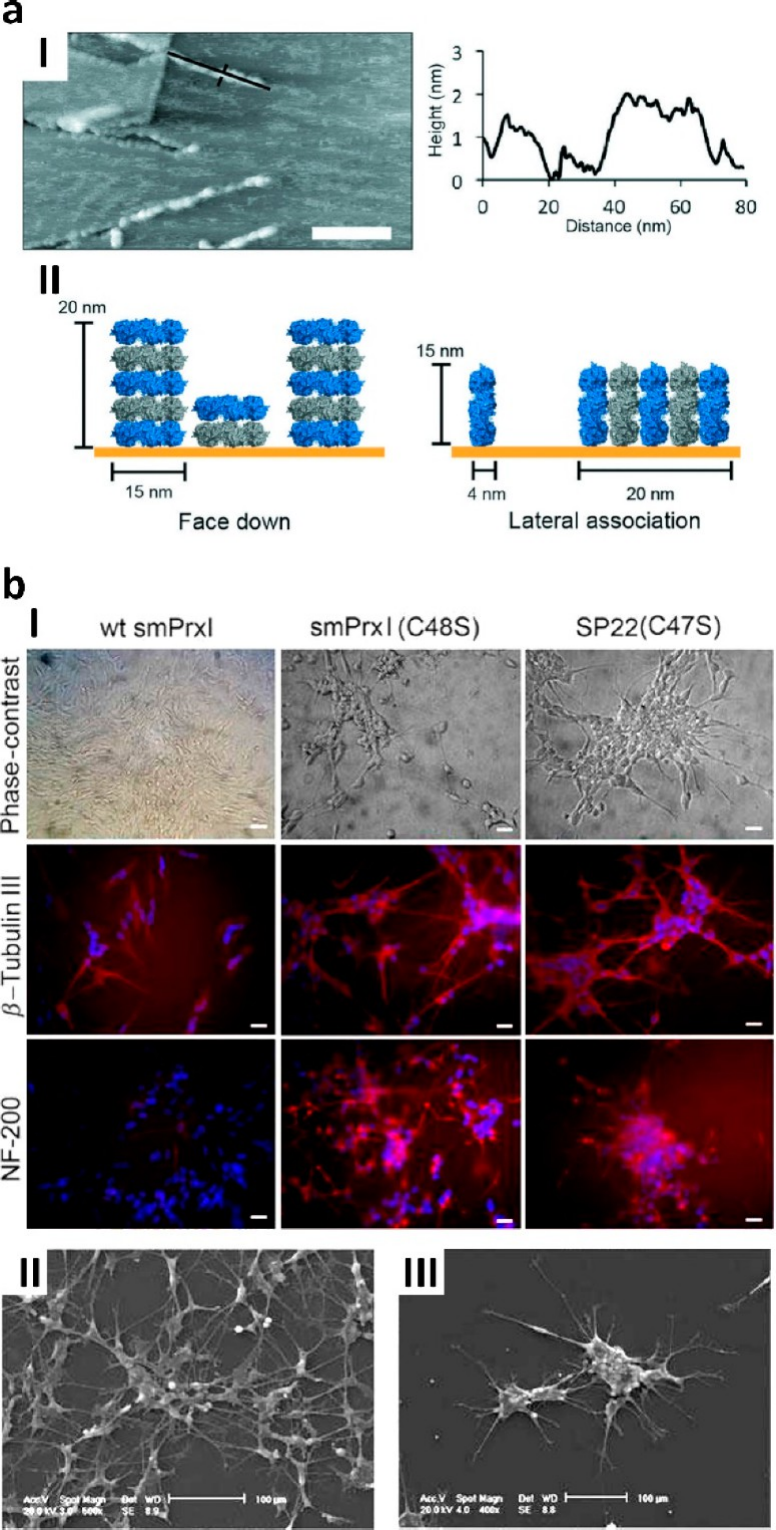
Gold-tethered Prx tubes and scaffolds for tissue engineering. (a) 2D flat assemblies of N-terminal polyHis-tagged hPrxIII tubes on gold layers (I, scale bar = 50 nm) adsorbed with face-on or side-on (lateral association) orientation (II). Adapted from ref (75), with the permission of AIP Publishing. (b) SH-SY5Y cells grown on substrates of N-terminal polyHis-tagged tubes from mutants Cys48Ser and Cys47Ser of *Sm*PrxI or *Bt*PrxIII, respectively, undergo morphological changes forming sprouting neurites as observed by PCM; conversely, no significant changes are observed when seeding cells over wild-type ring Prxs. The morphological changes are confirmed by IFM showing high expression of the neuronal markers NF200 and β-tubulin III as demonstrated by IFM, thus indicating differentiation toward neuronal-like cells; to note, cells tend to form large networks (I, scale bars = 15 μm). The neural-like phenotype and the cell networks are especially highlighted by electron micrographs with the formation of long sprouting neurites on both Cys48Ser (II) and Cys47Ser tube substrates (III). Adapted with permission from ref (95). Copyright 2016 John Wiley & Sons, Ltd.

Proteins supramolecular structures with preferential orientation on flat 2D gold are promising candidates to build ordered functional 2D or 3D assemblies for applications ranging from cell adhesion to biodevice engineering.^105^ In this regard, N-terminal polyHis-tagged hPrxIII tubes can be assembled from stacking of rings on flat gold surfaces with preferential orientation at pH 7.2. The protein adsorbs as single discrete rings at low concentration (see section 3.2.4) that, however, stack into tubes upon increasing at concentration 50 μg mL^–1^ in side-on (laterally) or face-on mode (Figure 13a).^75^ Furthermore, face-on adsorption is favored when coating the gold with SAM of 4-mercaptobenzoic acid (MBA) and attaching the protein by chemical cross-linking and, notably, by pretreating the protein at pH 4.0 resulting in long tubes, in agreement with other data (see section 3.1.2).^75^

The use of Prx tubes is not limited to the interaction with nonliving matter such as inorganic surfaces or NPs. Examples are the N-terminal polyHis-tagged *Sm*PrxI and *Bt*PrxIII whose mutants Cys48Ser and Cys47Ser are known to constitutively self-assemble into long tubes of stacked rings^38,62^ to be used as biocompatible substrates for cells. Namely, human neuroblastoma SH-SY5Y can be seeded and differentiated into neuronal-like phenotypes sprouting neurites and forming a cell network without the addition of N2, as shown by phase contrast microscopy (PCM) and confirmed by expression of neural markers β-tubulin III and NF-200. The differentiation is observed on both Prx-based substrates while being almost absent when seeding the cells over the wild-type proteins forming single rings (Figure 13b).^95^ To note, very similar effects are observed if using neural cancer stem cells (NCSCs) from human glioblastoma which are prone to adhesion of the neurospheres before spreading and differentiation on both the Prx tubes.^95^ Though the mechanism resulting is still under investigation, these data suggest that the sole tube-like architecture is mandatory to trigger the differentiation pathway whatever the Prx used, likely supporting these materials as universal tissue-free proteinaceous scaffolds for tissue engineering.

Protein-based self-assembling scaffolds are increasingly gaining interest as favorable and efficient scaffolds in tissue engineering applications as they can self-assemble under mild conditions into supramolecular structures mimicking the native extracellular matrix.^119^

## 4 Conclusions and Future Perspectives

The expanding universe of protein-based nanotechnology is currently flowing through the growing field of applied nanoscience, with broad impacts on nearly economic sectors, from electronics to energy, biomedicine, cosmetics, defense, automotive, and agriculture. Indeed, the global nanotechnology market is expected to exceed US $125 billion by 2024,^120^ thus advancing technology through increasing funding resources for R&D activities, miniaturization of devices, and strategic alliances between countries. Analysis of expanding market trends show that electronics, energy, and biomedicine account for over 70% of the growth, with nanoparticles, nanolithography, and nanodevices being the most significant.

This review aimed at highlighting that the structural and biochemical plasticity of Prxs can be also exploited across different multidisciplinary areas of applied science from electronics to biomedicine. The reported examples clearly demonstrate that control over the assembly/disassembly of Prxs into supramolecular complexes and their interactions with inorganic and organic materials can be easily achieved under mild experimental conditions *in vitro*. This is augmented using genetic engineering and/or chemical strategies: (1) presence/absence and length of N-terminal polyHis tags, (2) presence/absence of divalent metal cations and metal chelators, (3) pH changes, (4) reducing/oxidizing conditions, (5) amino acid mutations, (6) ionic strength, (7) protein concentration, (8) SAM-coated surfaces, and (9) design of Prx-derived peptides. Interestingly, in most cases changing these factors facilitates tuning the morpheein behavior and the interaction with nanomaterials. Evidence is presented for how to harness the oligomeric transitions of Prxs in solution from rings^29,38,46,70,75,78−80^ to hollow colloidal and surface-tethered tubes^29,38,46,70,75,80^ as well as cage-like particles and catenanes.^29,53^ Studies also show methods to improve such complexes making them suitable tectons for building nanostructures such as 2D protein arrays^41^ and nanoribbon-based 3D hydrogels.^89^ Their derivatives can be coupled to optoelectronic compounds for building transistor devices,^90^ 0D and 1D arrays of colloidal metal and mineralized NPs with electrical polarizability^46,94^ and 1D SERS-active metal NPs clusters.^84^ The “sticky” features of the Prx rings have been shown to be useful for assembling metal NP-doped 3D GO materials^77,91^ and improving the stiffness and biological effects of rGO to induce growth and differentiation of tumor-derived cells^93^ as well as to obtain 2D plasmonic nanopores over graphene-coated solid-state membranes.^92^ Finally, bare tubes of stacked Prx rings have been demonstrated to be efficient biological scaffolds for adhesion and differentiation of stem and tumor-derived cells with no need of differentiating supplements.^95^ The use of Prx for bionanotechnology purposes is just initiated and more efforts should be made in the future to completely master its morpheein behavior in order to easily access all the oligomeric structures available for Prx. For example, the interfaces involved in the Prx’s nanocage structure are not determined at the atomic level, even though some indications on how to stabilize this array already exist; the employment of this supramolecular oligomer for practical purposes is, at present, limited, but still very promising if one considers the many applications of ferritin-like or virus-like nanocages in biomedicine and nanotechnology reported so far.^121−123^ The valuable adaptability of such patterned protein complexes expands even more broadly if looking at other ring-shaped protein oligomers that do not belong to the Prx family.^48,49,72−74,124−138^ Taken together, all these features make protein morpheeins with supramolecular structures suitable for hierarchical nanofabrication of structures and devices,^86,88^ a concept that is currently emerging to overcome the limitations and necessities inherent to the “top-down” and “bottom-up” strategies, i.e., the scaling down of the size limit of nanofabrication and improving the control over self-assembling structures to create nanometric objects with collective behavior and coupling phenomena with augmented abilities to interact with light and biological matter.^139,140^
